# New Routes to Phylogeography: A Bayesian Structured Coalescent Approximation

**DOI:** 10.1371/journal.pgen.1005421

**Published:** 2015-08-12

**Authors:** Nicola De Maio, Chieh-Hsi Wu, Kathleen M O’Reilly, Daniel Wilson

**Affiliations:** 1 Institute for Emerging Infections, Oxford Martin School, Oxford, United Kingdom; 2 Nuffield Department of Medicine, University of Oxford, Oxford, United Kingdom; 3 MRC Centre for Outbreak Analysis and Modelling, School of Public Health, Faculty of Medicine, Imperial College London, London, United Kingdom; 4 Wellcome Trust Centre for Human Genetics, University of Oxford, Oxford, United Kingdom; Stanford University, UNITED STATES

## Abstract

Phylogeographic methods aim to infer migration trends and the history of sampled lineages from genetic data. Applications of phylogeography are broad, and in the context of pathogens include the reconstruction of transmission histories and the origin and emergence of outbreaks. Phylogeographic inference based on bottom-up population genetics models is computationally expensive, and as a result faster alternatives based on the evolution of discrete traits have become popular. In this paper, we show that inference of migration rates and root locations based on discrete trait models is extremely unreliable and sensitive to biased sampling. To address this problem, we introduce BASTA (BAyesian STructured coalescent Approximation), a new approach implemented in BEAST2 that combines the accuracy of methods based on the structured coalescent with the computational efficiency required to handle more than just few populations. We illustrate the potentially severe implications of poor model choice for phylogeographic analyses by investigating the zoonotic transmission of Ebola virus. Whereas the structured coalescent analysis correctly infers that successive human Ebola outbreaks have been seeded by a large unsampled non-human reservoir population, the discrete trait analysis implausibly concludes that undetected human-to-human transmission has allowed the virus to persist over the past four decades. As genomics takes on an increasingly prominent role informing the control and prevention of infectious diseases, it will be vital that phylogeographic inference provides robust insights into transmission history.

## Author Summary

When studying infectious diseases it is often important to understand how germs spread from location-to-location, person-to-person, or even one part of the body to another. Using phylogeographic methods, it is possible to recover the history of spread of pathogens (or other organisms) by studying their genetic material. Here we reveal that some popular, fast phylogeographic methods are inaccurate, and we introduce a new more reliable method to address the problem. By comparing different phylogeographic methods based on principled population models and fast alternatives, we found that different approaches can give diametrically opposed results, and we offer concrete examples in the context of the ongoing Ebola outbreak in West Africa and the world-wide outbreaks of Avian Influenza Virus and Tomato Yellow Leaf Curl Virus. We found that the most popular phylogeographic method often produces completely inaccurate conclusions. One of the reasons for its popularity has been its computational speed, which has allowed users to analyse large genetic datasets with complex models. More accurate approaches have until now been considerably slower, and therefore we propose a new method called BASTA that achieves good accuracy in a reasonable time. We are relying more and more on genetic sequencing to learn about the origin and spread of infections, and as this role continues to grow, it will be essential to use accurate phylogeographic methods when designing policies to prevent or curb the spread of disease.

## Introduction

Phylogeographic methods aim to infer many aspects of population evolution from genetic data. The phylogeography term often encompasses methods that infer changes in population size (phylodynamics) and population divergence events (see [1]). In this work, we focus on the inference of migration between distinct subpopulations (such as in the structured coalescent, see [2, 3]). For many years, nested clade phylogeographic analysis (NCPA, see e.g. [4, 5]) was the leading method to test for isolation and migration (reviewed in [1, 6]). More recently, model-based inference for phylogeography has flourished and has replaced NCPA as the new standard approach (reviewed in [7, 8]).

Probabilistic model-based inference for phylogeography has widely been used to study the spread of pathogens between geographic locations and to identify their original source [9–12], and they are commonly applied to study the migration history of animals [13–15], plants [16, 17], and even languages [18]. Phylogeographic methods are useful for addressing a wide range of questions in epidemiology, for example in studying transmission of pathogens between body compartments within a host [19], between individual hosts [20], between host social groups [21],and between host species [22].

One major class of modelling approaches comprise likelihood-based methods implementing the structured coalescent [23–27], which corresponds to the classic migration matrix model [28], a generalization of Wright’s Island model [29]. These approaches use the structured coalescent to infer migration rates and effective population sizes. However, they are impractical in scenarios with large numbers of subpopulations and migration events due to their computational demand. This is because they explore not only the parameters of primary interest (such as migration rates, population sizes, and phylogeny) but also all possible migration histories, vastly increasing the computational complexity.

Recently, an alternative phylogeographic approach has risen in prominence, which treats the migration of lineages between locations as if the location were a discrete trait, evolving in a manner analogous to the substitution of alleles at a genetic locus [9, 10, 15]. Since migration is modelled like mutation, this approach is referred to as “Mugration” by [30, 31], or “discrete trait analysis” (DTA in the following). The gained popularity of this approach (see e.g. [1]) is at least partly attributable to its computational efficiency and user-friendly software. However, the DTA model inherits a set of assumptions appropriate for the independent mutation of loci within lineages, but profoundly at odds with classical population genetics models of migration (see e.g. [3, 32, 33]), as summarised in Table A in S1 Text. While methods based on the structured coalescent, which explicitly accounts for the effects of migration on the shape and branch lengths of the genealogy, are in theory often preferable to DTA, the latter is frequently chosen due to the computational demands of current implementations of the structured coalescent. DTA is also commonly used to describe the evolution of discrete phenotypes. In many such cases, DTA is appropriate [34, 35]. However, use of the DTA entails a number of assumptions that are unusual or inappropriate when applied to the migration of lineages between geographic locations, for example (i) the relative size of subpopulations drifts over time, such that subpopulations can become lost (extinct) or fixed (the sole remaining subpopulation) instead of being constrained, e.g. by local competition, (ii) sample sizes across subpopulations are proportional to their relative size.

There is a scarcity of studies in the scientific literature assessing the accuracy of DTA and comparing different phylogeographic approaches, but concerns have been raised because, among other issues, DTA is thought to be sensitive to local sampling intensity [12, 36]. Further, the conceptual separation of coalescent process and migration process made by DTA is expected to lead to suboptimal use of information. Here we demonstrate that these concerns are well founded, in that DTA suffers from various biases and statistical inefficiency despite its computational speed.

To address the problems with DTA we introduce a new model-based approach that achieves a close approximation to the structured coalescent (similar in spirit to [37, 38]). The idea behind this approximation is to efficiently integrate over all possible migration histories, therefore reducing the computational effort needed to explore the parameters of primary interest. We implement this approach, called BASTA (BAyesian STructured coalescent Approximation), in the Bayesian phylogenetic package BEAST2 [31]. We compare its performance to DTA and MultiTypeTree (MTT, a recent Bayesian structured coalescent software, see [27]) using simulations based on the structured coalescent.

We illustrate the use of the method to reconstruct transmission dynamics in human, animal and plant viruses. We demonstrate the important influence of model choice on study conclusions through an analysis of genomic data from previous and ongoing Ebola epidemics [39] using different phylogeographic approaches to interpret the role of zoonotic events in the origin of human outbreaks. Our results show, based both on simulations and real data analyses, that DTA and structured coalescent methods can lead to different conclusions, and that DTA is often inaccurate.

## Methods

### The Structured Coalescent

In this section we define the structured coalescent before describing the DTA model and introducing BASTA. The structured coalescent is a statistical model describing the genealogy of individuals sampled from a structured population that evolves according to the migration matrix model [28]. For simplicity, here we assume individuals are haploid, but the model applies more generally. The key assumptions are: (i) The subpopulations, or *demes*, are stable in size over time, with their effective sizes defined by the vector ***θ***. (ii) Migration occurs at a constant rate over time, defined by the migration matrix ***f***, such that *f*_*a*,*b*_ is the total rate of migration of individuals from deme *a* to *b*, divided by the effective number of individuals in deme *a*. (iii) There is no substructure within demes. (iv) There are no differences in fitness between individuals. (v) Within demes, individuals are sampled at random. However, no assumptions are made about the total sample size nor the relative sample sizes per deme.

A potential source of confusion arises from the convention of using the *backwards-in-time* migration rate matrix ***m*** in the structured coalescent, defined such that *m*_*b*,*a*_ is the total rate of migration of individuals from deme *a* to *b*, divided by the effective number of individuals in deme *b*. Mathematically, *m*_*b*,*a*_ = *f*_*a*,*b*_ *θ*_*a*_/*θ*_*b*_. The backwards migration matrix ***m*** is considered convenient because it provides the rate at which a lineage appears to move between demes *backwards* in time. For this reason, we refer to ***f*** as the forwards-in-time migration rate matrix.

In the notation of [27], the demes are represented by a set *D*, sampled individuals are represented by the set *I*, the aligned sequences by the set *S* = {*s*_*i*_∣*i* ∈ *I*}, the sampling dates by the set *t*_*I*_ = {*t*_*i*_∣*i* ∈ *I*} and the sampling locations by the set *L* = {*l*_*i*_∣*i* ∈ *I*}. In addition to the parameters of primary interest, ***m*** and ***θ***, *T* represents the genealogy, ***μ*** the nucleotide substitution rate matrix and *M* the migration history of lineages in the tree, i.e. the timing, source, sink and lineage involved in each migration event.

MultiTypeTree (MTT) is a method implemented in BEAST2 for estimating the parameters of the structured coalescent by Bayesian inference [27]. Formally, the target of inference is the posterior distribution of the parameters given the data:

$$P\left(T,M,\mu ,m,\theta |S,t_{I},L\right)\propto P\left(S|T,t_{I},\mu \right)P\left(T,M|t_{I},L,m,\theta \right)P\left(\mu ,m,\theta \right).$$ (1)

The posterior consists of several components. The first term on the right is the likelihood of the sequences given the genealogy and substitution model, which is computed using Felsenstein’s pruning algorithm [40]. The second term is the probability density of the genealogy and migration history under the structured coalescent given the migration matrix and effective population sizes. The third term represents the prior distribution assumed for the parameters, and might be factored into independent priors for the separate parameters, *P*(***μ***)*P*(***m***)*P*(***θ***).

To calculate *P*(*T*,*M*∣*t*_*I*_,*L*,***m***,***θ***) under the structured coalescent, the sequence of *B* time intervals between successive events (coalescence, sampling, or migration) is considered, starting from the most recent sample and going back to the root of the genealogy. Suppose that the vector ***τ*** records the duration of each time interval. For a haploid population,

$$P\left(T,M|t_{I},L,m,\theta \right)=\prod_{i=1}^{B}L_{i},$$ (2)

where

Li=exp[-τi∑d∈D((ki,d2)1θd+ki,d∑d′∈D,d′≠dmdd′)]Ei, (3)

*k*_*i*,*d*_ is the number of lineages in deme *d* in interval *i*, and *E*_*i*_ is the contribution of the event that ends interval *i*:

$$E_{i}=\{\begin{array}{cc} 1 & \text{if}\;\text{it}\;\text{is}\;\text{a}\;\text{sampling}\;\text{event}\\ m_{dd^{\prime }} & \text{if}\;\text{it}\;\text{is}\;\text{a}\;\text{migration}\;\text{event}\;\text{from}\;d\;\text{to}\;d^{\prime }\\ \frac{1}{\theta_{d}} & \text{if}\;\text{it}\;\text{is}\;\text{a}\;\text{coalescence}\;\text{event}\;\text{in}\;\text{deme}\;d\text{.} \end{array}$$ (4)

### Discrete Trait Analysis

**Fig 1:**
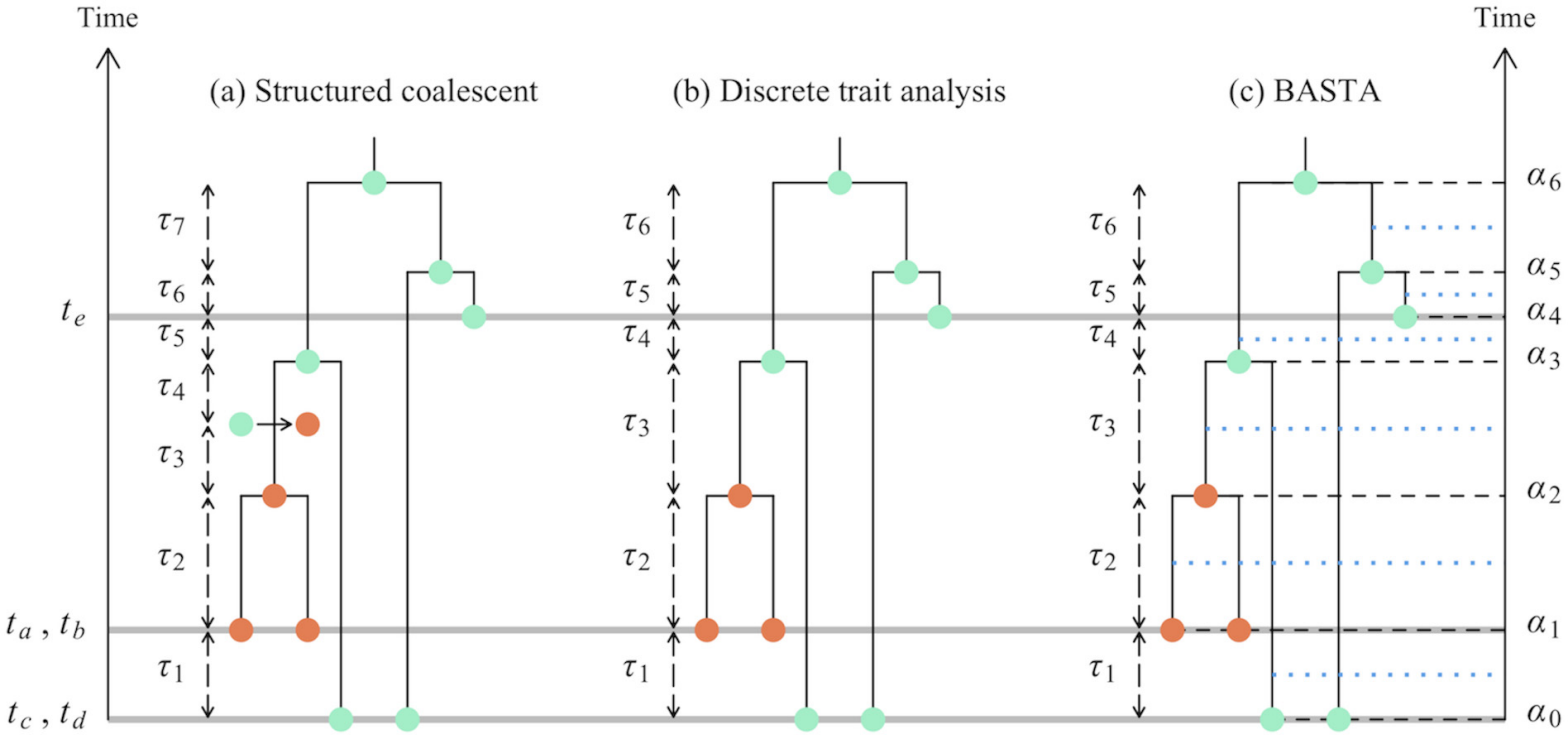
Graphical representation of phylogeographic models. In this study we consider three phylogeographic methods: the structured coalescent, DTA, and BASTA. This figure shows some of the differences in these models, in particular in the modelled events and time intervals. Coloured dots show different subpopulations (one orange subpopulation and one turquoise for both sampled and internal nodes in the genealogy. a) In the structured coalescent eight events are considered, delimiting seven time intervals of lengths *τ*_1_…*τ*_7_. Three of these events are sampling events (denoted by the grey horizontal lines), one is a migration event (represented by an arrow between two coloured dots), and four are coalescence events. b) In DTA, migration events are not explicitly parameterized, so we have a total of seven sampling or coalescence events, delimiting six time intervals of lengths *τ*_1_…*τ*_6_. While locations for internal nodes are depicted in the figure, the method effectively integrates over all possible ancestral locations at each MCMC step. c) As in DTA, BASTA does not consider migration events, and therefore has seven events and six time intervals. Yet, each of these intervals is split exactly in half (blue horizontal dotted lines), and the two halves are considered separately. Again, as in DTA, at each MCMC step BASTA integrates over all possible internal nodes locations.

In the structured coalescent, migration events affecting lineages in the genealogy are explicitly parameterized and estimated (Fig 1). In the context of migration, DTA [9, 15] is a model that achieves much greater computational efficiency by integrating over all possible migration histories using the pruning algorithm [40], which is widely-used in phylogenetics to integrate over all possible mutation histories. However, treating the migration process as if it were analogous to a mutation process implies a set of assumptions that differ substantially from standard migration models. Namely: (i) The total effective population size is fixed to *θ*, but demes can change in relative size over time due to drift (the chance birth and death of individuals); the rate of drift is the same across demes and determined by the total effective population size. (ii) Demes can also change in relative size due to migration, which occurs at a constant rate per lineage, defined by the forwards-in-time migration matrix ***f***. (iii) Individuals are sampled at random from demes in proportion to their relative size.

There are some unusual consequences of the DTA modelling assumptions: (i) Demes can be lost, and they can be resurrected. (ii) The relative sampling intensities of the demes are treated as data, informative about the migration parameters, even before any sequence data is analysed. (iii) It is unclear what the relationship is between the effective population size parameter *θ* of the DTA and the vector of effective population sizes ***θ*** of the structured coalescent, hindering interpretation.

Formally, the target of inference in a Bayesian implementation of DTA is the posterior distribution of the parameters given the data. This differs considerably from Eq 1 of MTT:

$$P\left(T,\mu ,f,\theta |S,t_{I},L\right)\propto P\left(L|T,t_{I},f\right)P\left(S|T,t_{I},\mu \right)P\left(T|t_{I},\theta \right)P\left(\mu ,f,\theta \right).$$ (5)

The sampling locations *L* are treated as informative data, rather than uninformative auxiliary variables. The first term on the right is the likelihood of the sampling locations given the genealogy and migration matrix, calculated by integrating over all possible migration histories using the pruning algorithm. The second term is the likelihood of the sequences integrated over all possible mutation histories using the pruning algorithm, as in MTT. The third term is the probability density of the genealogy, approximated by a standard neutral coalescent prior for an unstructured population [41]. The fourth term represents the prior distribution. A different prior is required for the effective population size parameter *θ* in DTA because, differently from MTT, *θ* is the same for all demes.

In essence, the assumptions of DTA are well motivated when employed to analyse randomly sampled alleles or discrete phenotypes which evolve independently across individuals. But they are questionable when employed to analyse the migration of individuals between subpopulations, whose relative frequencies are maintained by external forces such as resource availability, and for which the sampling frame might not be related to the relative sizes of the subpopulations.

The consequences of the various approximations that the DTA represents have not been thoroughly explored in the literature, despite the popularity of the approach. One concern is that the assumption that sampling intensity is proportional to subpopulation size leads to biased estimates of migration rates when this assumption is not met [12, 36]. Second, ignoring the population structure when calculating the probability of the coalescent tree could lead to bias or lost power. For example, when migration rates are very low, one expects very long branches close to the root. This interdependency between the shape and branch lengths of the genealogy and the migration process is ignored by DTA, which could reduce accuracy. We test these concerns using simulations, described later in the Methods.

### BASTA

As an alternative to DTA, we pursue an approximation to the structured coalescent that is both accurate and computationally efficient, which we have developed in a Bayesian statistical framework and implemented in BEAST 2, a software package for Bayesian evolutionary analysis. Like DTA, we gain computational efficiency by integrating over all possible migration histories with an approximation. Unlike DTA, we treat the genealogy as informative about the migration process and the sampling locations as uninformative a priori.

In our approximation to the structured coalescent, we split each interval between successive coalescence events into two sub-intervals, within which the migration of lineages (backwards-in-time) is independent of one another (similarly to [37, 38]). This approximation differs from the assumptions of DTA because it ensures that the rate of coalescence between lineages depends on the probability they are in the same deme at the same time. It is approximate because (i) within each interval we model locations of lineages independently of each other, and (ii) we update the probability distribution of lineages among demes only at the beginning and end of each interval instead of continuously in time.

Formally, we seek to approximate the same posterior distribution as MTT, but integrated over all possible migration histories:

$$P\left(T,\mu ,m,\theta |S,t_{I},L\right)\propto P\left(S|T,t_{I},\mu \right)P\left(T|t_{I},L,m,\theta \right)P\left(\mu ,m,\theta \right).$$ (6)

The first term on the right is the likelihood of the sequences given the genealogy and substitution model, as in Eq 1. The second term is the probability density of the genealogy under the structured coalescent, integrated over migration histories. This must be approximated in BASTA because no exact form is known. The third term represents the prior distribution for the parameters, as in Eq 1.

To approximate *P*(*T*∣*t*_*I*_,*L*,***m***,***θ***) in BASTA, we consider the probability density of each time interval between successive events (coalescence or sampling). Denoting each interval *A*_*i*_ = [*α*_*i* − 1_,*α*_*i*_], where *α*_*i*_ is the older event time of *A*_*i*_ and *α*_*i* − 1_ the more recent one, the probability density of interval *A*_*i*_ can be written as

$$L_{i}^{\prime }=\exp[-\int_{\alpha_{i-1}}^{\alpha_{i}}\sum_{d\in D}\sum_{l\in \Lambda }\sum_{l^{\prime }\in \Lambda ,l^{\prime }\neq l}P(d_{l}=d,d_{l^{\prime }}=d|t)\frac{1}{\theta_{d}}dt]E_{i}^{\prime },$$ (7)

where Λ is the set of all extant lineages during interval *A*_*i*_, *d*_*l*_ is the deme to which lineage *l* belongs, and *P*(*d*_*l*_ = *d*,*d*_*l*′_ = *d*∣*t*) is the probability that lineages *l* and *l*′ are in the same deme *d* at time *t*. $E_{i}^{\prime }$ is the contribution of the coalescent or sampling event:

$$E_{i}=\{\begin{array}{cc} 1 & \text{if}\;\text{it}\;\text{is}\;\text{a}\;\text{sampling}\;\text{event,}\\ \sum_{d\in D}P_{l,\alpha_{i},d}P_{l^{\prime },\alpha_{i},d}\frac{1}{\theta_{d}} & \text{if}\;\text{it}\;\text{is}\;\text{a}\;\text{coalescence}\;\text{between}\;l\;\text{and}\;l^{\prime }\text{.} \end{array}$$ (8)

To approximate $L_{i}^{\prime }$ we first substitute *P*(*d*_*l*_ = *d*,*d*_*l*′_ = *d*∣*t*) with *P*(*d*_*l*_ = *d*∣*t*)*P*(*d*_*l*′_ = *d*∣*t*), which treats the migration of lineages as if they were independent of one another. As shorthand, we define ***P***_*l*,*t*_ to be the vector whose *d*th element is *P*_*l*,*t*,*d*_ = *P*(*d*_*l*_ = *d*∣*t*). Next, we split each interval *A*_*i*_ into two sub-intervals of equal length *A*_*i*1_ = [*α*_*i* − 1_,(*α*_*i*_+*α*_*i* − 1_)/2] and *A*_*i*2_ = [(*α*_*i*_+*α*_*i* − 1_)/2,*α*_*i*_], and replace ***P***_*l*,*t*_ with ***P***_*l*,*α*_*i* − 1__ for all *t* in *A*_*i*1_ and ***P***_*l*,*α*_*i*__ for all *t* in *A*_*i*2_. The approximated probability density contributions of *A*_*i*1_ and *A*_*i*2_ become:

$${\tilde{L}}_{i1}=\exp[-\frac{\tau_{i}}{2}\sum_{d\in D}\sum_{l\in \Lambda }\sum_{l^{\prime }\in \Lambda ,l^{\prime }\neq l}P_{l,\alpha_{i-1},d}P_{l^{\prime },\alpha_{i-1},d}\frac{1}{\theta_{d}}]$$ (9)

and

$${\tilde{L}}_{i2}=\exp[-\frac{\tau_{i}}{2}\sum_{d\in D}\sum_{l\in \Lambda }\sum_{l^{\prime }\in \Lambda ,l^{\prime }\neq l}P_{l,\alpha_{i},d}P_{l^{\prime },\alpha_{i},d}\frac{1}{\theta_{d}}]E_{i}^{\prime }.$$ (10)

Further improvements to the approximation could be obtained by considering more sub-intervals, albeit at increased computational cost.

Between intervals, the probability distribution of lineages among demes is updated as

$$P_{l,\alpha_{i}}=P_{l,\alpha_{i-1}}\exp(\tau_{i}\cdot m),$$ (11)

where time is scaled in *N*_*e*_ = ∑_*d* ∈ *D*_ *θ*_*d*_ generations, the exponential is a matrix exponential, and ***m*** is the backwards-in-time migration rate matrix, whose diagonal elements are defined such that the rows sum to zero. For a lineage *l* sampled from deme *d* at time *t*, ***P***_*l*,*t*_ is a vector whose *d*th element equals one and all other entries equal zero. If lineages *l*_1_ and *l*_2_ coalesce to an ancestral lineage *l* at time *t*, then

$$P_{l,t}=\frac{\left(\frac{P_{l_{1},t,1}P_{l_{2},t,1}}{\theta_{1}},\ldots ,\frac{P_{l_{1},t,\left|D\right|}P_{l_{2},t,\left|D\right|}}{\theta_{\left|D\right|}}\right)}{\sum_{d=1}^{\left|D\right|}\frac{P_{l_{1},t,d}P_{l_{2},t,d}}{\theta_{d}}},$$ (12)

which is the normalised entrywise product (element by element product) of the distributions of the coalescing lineages.

The probability density of the genealogy under the structured coalescent, integrated over migration histories, is finally approximated as

$$P\left(T|t_{I},L,m,\theta \right)=\prod_{i=1}^{B}{\tilde{L}}_{i1}{\tilde{L}}_{i2}.$$ (13)

Details of how we efficiently compute these quantities, in particular Eq 10, are given in S1 Text. The software implementing BASTA can be freely downloaded from https://bitbucket.org/nicofmay/basta-bayesian-structured-coalescent-approximation, including the source code. The software can alternatively be installed from the graphical user interface BEAUti [42] of BEAST2. Example files and data from the analyses described hereby can be found in Supplementary S1 Dataset.

### Simulations

To assess the adequacy of the approximations in BASTA, and to compare its performance to MTT and DTA, we performed simulations under the structured coalescent [2, 3] with the software msms [43]. We quantified the performance of the methods by analysing a large number of datasets simulated from a range of migration rates. By comparing the simulated (“true”) and estimated parameters, we could assess performance using a number of statistics:

**Bias**: mean difference between the simulated and estimated parameter.
**RMSE**: square root of the mean squared difference between the simulated and estimated parameter.
**Correlation**: Pearson’s correlation coefficient between the simulated and estimated parameter.
**Calibration**: proportion of datasets for which the simulated parameter lay within the 95% credible interval.

In all cases, point estimates were taken to be the estimated posterior median and 95% credible intervals were taken to be the 95% region of the estimated posterior distribution with the highest density. The theoretically optimal values for the bias, RMSE and correlation are 0, 0 and 1 respectively. The theoretically optimal value for the calibration is 0.95 when the parameters are simulated under the same prior distribution as that used for analysis. Values greater than 0.95 are considered conservative.

Since we expect the information content of the sequences (including sequence length and diversity) to have a strong effect on the analysis, we investigated three levels of genetic information:

**Fixed tree**: abundant genetic data so that the genealogical topology and branch lengths are essentially known (up to a scaling factor) without error, achieved by providing BEAST2 with the simulated genealogy. Even in this scenario, we still expect uncertainty in parameter estimates due to inherent stochasticity in the migration process.
**Variable tree**: limited genetic data so that there is uncertainty in the genealogy. For this we simulated an alignment of 2000 bp using SeqGen [44] with a transition/transversion ratio of *κ* = 3 and mutation rate of 0.01 in units of *N*_*e*_ generations, and we estimated the genealogy in BEAST2 along with the other parameters.
**No data**: to test for susceptibility to sampling bias, we took the unusual step of analysing sequence data that were completely uninformative about the genealogy by providing a single ambiguous base (‘N’) for each individual. Unless the method is biased, the posterior produced by BEAST2 in this case should equal the prior.

We simulated under two scenarios, a “Continental” model with two subpopulations, and an “Archipelago” model with eight subpopulations, and investigated the performance of the methods under different sampling strategies (even versus uneven) and mean migration rate (fast versus slow).

In the Continental model, we considered two subpopulations, with different rates of migration between the two, and a total sample size of 200. We compared even sampling, in which 100 individuals were sampled per subpopulation, to uneven sampling, in which 10 individuals were sampled from one subpopulation and 190 from the other. We sample migration rates used in simulations from the DTA prior distribution, that is, relative migration rates *r*_1,2_ and *r*_2,1_ were simulated from independent Γ(1.0,1.0) distributions. This mildly favours DTA because MTT and BASTA use log-normal priors with *σ* = 4 instead. The relative migration rates were then rescaled so that the mean migration rate $\bar{f}$ was equal to a value simulated from an exponential distribution with mean 0.1 (very slow), 0.5 (slow), 2.0 (moderate) or 5.0 (fast). After rescaling, *f*_1,2_ = *c* *r*_1,2_ and *f*_2,1_ = *c* *r*_2,1_, where $c=\bar{f}(r_{1,2}+r_{2,1})/(2\;r_{1,2}\;r_{2,1})$.

Since DTA assumes that the rate of drift is the same in every deme, we fixed all effective population sizes in the simulations and in BEAST2 to be equal to one in order to reduce the disparity in modelling assumptions. This has the effect of (i) simplifying interconversion between forwards-in-time and backwards-in-time migration rates, so that *f*_*i*,*j*_ = *m*_*j*,*i*_ and (ii) scaling migration rates in “coalescent time units”. However, the interpretation of effective population size (and hence coalescent time units) differs between the structured coalescent and DTA models, so we based model comparison on the relative migration rate *f*_1,2_/*f*_2,1_.

In the Archipelago model, we considered two groups (archipelagos) of four subpopulations (islands), with two migration rates: a faster rate between islands in the same archipelago and a slower rate between islands not in the same archipelago. Forty individuals were sampled from each subpopulation. We fixed the rate of migration within (*f*_*w*_) and between (*f*_*b*_) archipelagos to *f*_*w*_/*f*_*b*_ = 10 and simulated *f*_*b*_ from an exponential distribution with mean 0.5.

From all simulations, migration rates and root location were then estimated using DTA [9, 15], MTT and BASTA, all as implemented in BEAST2. For the “No data” scenario, the posteriors from ten independent chains were merged, each of 5 × 10^6^ iterations. For the “Fixed tree” scenario, a single chain of respectively 10^6^, 2 × 10^5^, and 10^5^ iterations for DTA, MTT and BASTA was used. For the “Variable tree” scenario, we used a single chain of respectively 2 × 10^7^, 2 × 10^7^, and 10^7^ iterations for DTA, MTT and BASTA. Finally, for the “Archipelago” scenario we used a single chain of 2 × 10^6^ iterations for both MTT and BASTA.

### Avian Influenza Virus and Tomato Yellow Leaf Curl Virus Datasets

We applied DTA, MTT and BASTA to two datasets with moderately high numbers of subpopulations, one consisting of a collection of Avian Influenza Virus (AIV) haemagglutinin (HA) segments collected from different avian hosts and different locations [45], and one of a collection of Tomato Yellow Leaf Curl Virus (TYLCV) sequences (the CP dataset free of detectable recombination from [46]). For the AIV data we use two distinct subdivisions of samples into discrete host species classes, following the classifications in [45]. The first involves five groups, and the second ten groups. For the TYLCV dataset we used a single subdivision of samples into eight geographical classes, obtained following [46]. In these analyses, the effective population sizes of all demes were set equal in both MTT and BASTA.

### Ebola Transmission Study

To study changes of host type in Ebola we used whole genome Ebola sequences from 78 patients recently obtained and aligned with sequences from previous outbreaks [39]. The authors of this study investigated the phylogenetic relationship of samples within or between Ebola outbreaks. We applied the three phylogeographic methods presented above to infer the contribution of zoonotic events to Ebola spread. We used the same alignment provided in [39] for the BEAST2 analysis, including sampling dates, but we also added information regarding host type. We defined two subpopulations, human and animal reservoir, and we allowed lineages to transmit forwards in time from the animal reservoir to a human host, but not vice-versa. So our phylogeographic model had two locations (respectively human and animal reservoir) but migration was only assumed to occur in one direction. This results in a structured coalescent model with three phylogeographic parameters for MTT and BASTA (one migration rate and two effective population sizes), but only two parameters for DTA, as only a single general effective population size can be defined in that model. A peculiarity of these analyses is that no samples from one of the two considered populations were available. While this might seem an impassable limitation, previous studies have shown that the structured coalescent can provide meaningful estimates even in the absence of samples from one populations (i.e. “ghost deme”, see [47]), suggesting that it is possible to perform statistical inference of zoonosis rates in this scenario.

Since the inclusion of no animal samples is unusual, we considered a second, more typical, analysis in which we included genetic sequences from bats. Relatively little sequencing has been performed in potential animal reservoirs, so we were able to include only partial Ebola virus sequences from a 265 bp region of the polymerase (L) gene from seven bats collected in [48]. In this analysis, it was necessary to allow a small but non-zero rate of migration from humans to the animal reservoir to avoid predetermining inference of the ancestral location of the root. Therefore we constrained the migration rate from humans to animals at a rate 10^5^ times lower than the animal to human rate. This preserves the ability of the model to infer ancestral locations in either of the two subpopulations, once samples from the animal reservoir have been included.

## Results

### DTA is Inherently Biased by the Sampling Process

To test for susceptibility to biases associated with sampling strategy in DTA, MTT and BASTA, we analysed datasets completely lacking any genetic information (the “No data” scenario), and containing only the sampling locations of 200 individuals from two populations in our Continental model. We compared two sampling strategies. In the first, individuals were sampled evenly (100 per subpopulation) and in the second, unevenly (10 from one and 190 from the other). All three methods are Bayesian, so in the absence of information we expect the posterior distribution of the parameter of interest to be unchanged from the prior. For comparability across methods, the parameter we analysed was *f*_1,2_/*f*_2,1_, the ratio of migration rates between the two subpopulations.

**Fig 2:**
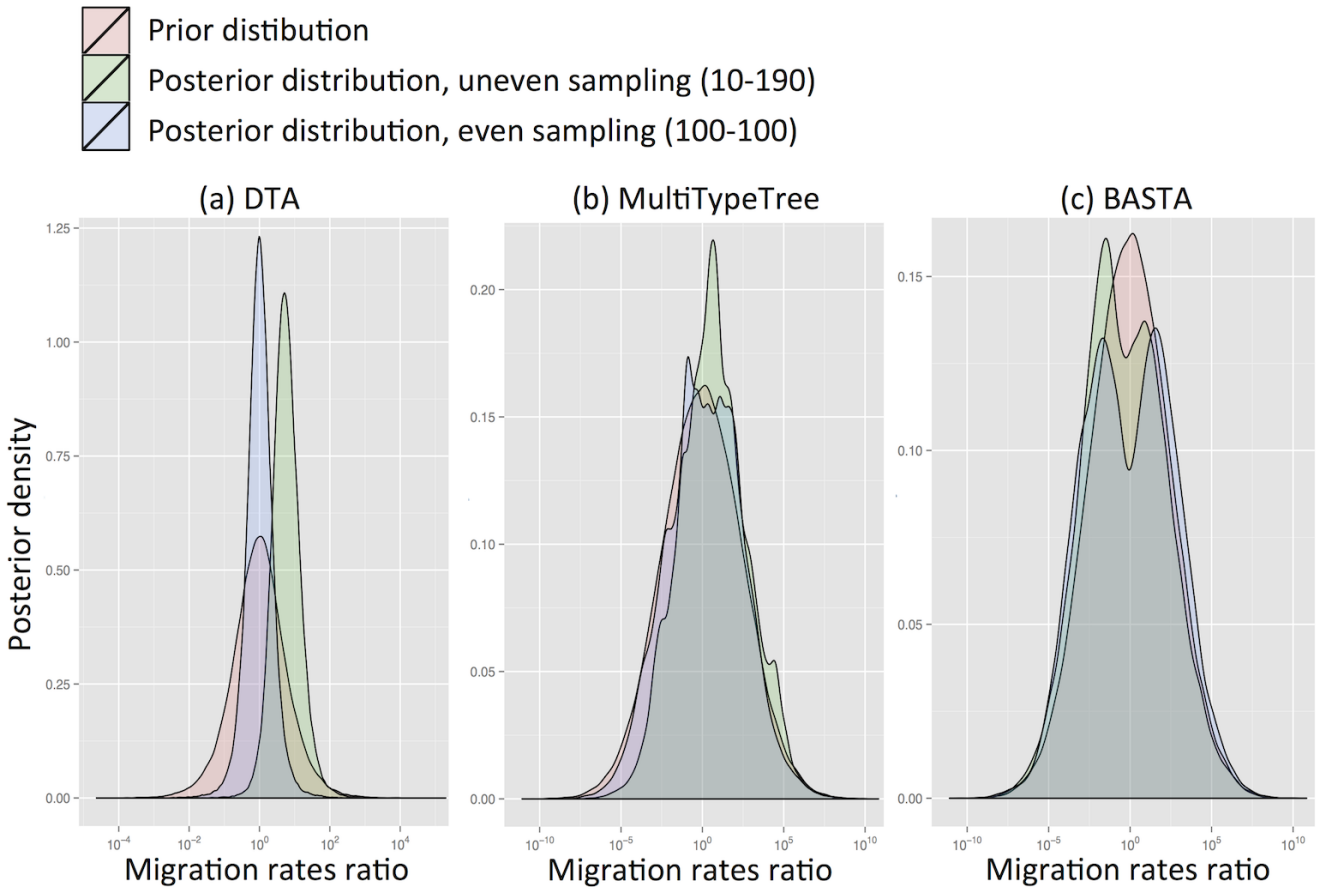
DTA is inherently biased by the sampling process. To test for inherent sampling bias, we analysed a dataset containing just sampling locations, but no genetic information using (a) DTA, (b) MTT and (c) BASTA. For a method robust to sampling, the posteriors (green and blue distributions) should be unchanged from the prior (pink distribution). However, DTA treats the sampling process as informative about migration parameters, unlike the structured coalescent-based methods, introducing a sampling strategy-dependent bias. The blue and green posterior distributions correspond respectively to even sampling (100 samples per subpopulation) and uneven sampling (10 and 190 samples per location). The mean migration rate was $\bar{f}=5.0$. Each plot is obtained from ten merged posteriors of independent MCMC runs each of 5 × 10^6^ iterations.

We found that for DTA the posterior distribution was substantially different to the prior, exhibiting a bias that depended on sampling, and a reduction in parameter uncertainty, unlike the structured coalescent methods (MTT and BASTA). Particularly with high migration rates (mean $\bar{f}=5.0$) DTA posteriors showed large biases (posterior median of rates log-ratio 1.7 with standard deviation 0.94, Fig 2a), indicating that the sampling strategy significantly influenced the result. The posterior distributions for MTT and BASTA were unbiased, centred on the prior mean of 0.0, but noticeably less smooth (Fig 2b and 2c), indicating that they need running for longer than DTA.

Even when migration rates were low (mean $\bar{f}=0.1$) DTA substantially over-estimated them (Fig. Aa in S1 Text). This is because the DTA model expects that, at low migration rates, one subpopulation will drift to high frequency, and that samples are collected proportionally to subpopulation size, so a random sample would be unlikely to capture multiple locations. The presence of multiple locations therefore suggests to DTA an appreciable migration rate. In contrast, the structured coalescent allows arbitrary sampling schemes and accounts for the fact that there must be at least *D* − 1 migration events when *D* subpopulations are sampled, regardless of migration rates.

### DTA Under-represents Uncertainty

Next we assessed the accuracy of the 95% credibility intervals produced by the three methods. Again employing the Continental model, this time we quantified the performance of the methods in the favourable situation of highly informative sequences. Methods are expected to perform best when genetic data is so informative that the phylogenetic tree can be estimated with little error. We investigated this scenario by providing the true tree topology and relative branch lengths as input, and estimating only the tree height together with the migration rate parameters (the “Fixed tree” scenario). As before, we analysed *f*_1,2_/*f*_2,1_ the ratio of migration rates between the two subpopulations.

**Fig 3:**
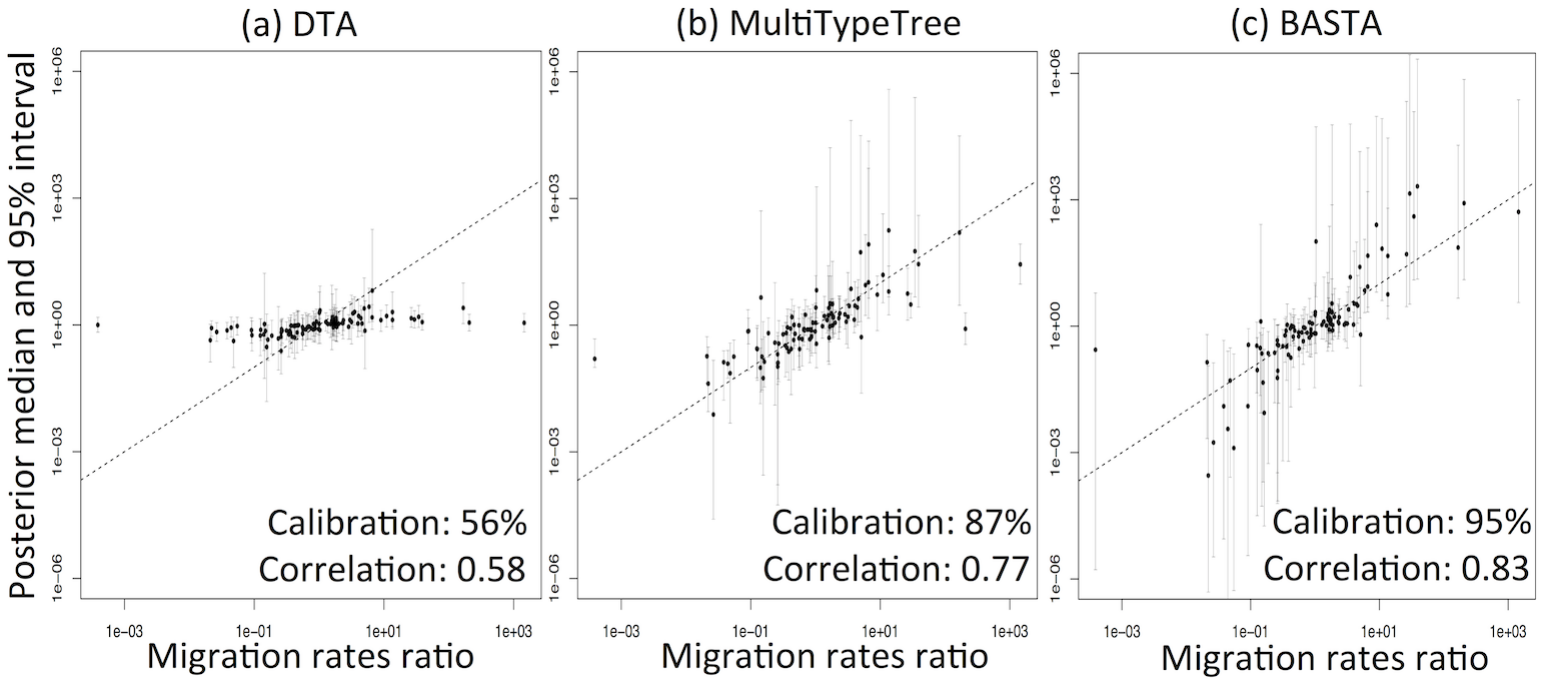
DTA under-represents uncertainty and lacks statistical efficiency. To test the accuracy of the 95% credible intervals produced by (a) DTA, (b) MTT and (c) BASTA, we simulated and analysed 100 datasets under the two-population “Continental” model with even sampling of 100 individuals per subpopulation. We provided the true genealogy to BEAST2, as if it were estimated without error; in this scenario methods are expected to give the best accuracy. The migration rates between the subpopulations were simulated for each dataset from a prior distribution, and we compared the “true” ratio *f*_1,2_/*f*_2,1_ (horizontal axis) to the point estimate (posterior median; vertical axis, points) and 95% credible interval (2.5 and 97.5 percentiles; error bars). The results show a weak correlation between the truth and the point estimates for DTA, compared to MTT and BASTA, indicating poor statistical efficiency. The percentage of datasets in which the 95% credible intervals contained the truth revealed that DTA was poorly calibrated compared to MTT, BASTA and the theoretical target of 95%. The mean migration rate was high ($\bar{f}=5.0$). The dashed line indicates the hypothetical optimal estimate. Number of MCMC steps for DTA, MTT and BASTA are respectively 10^6^, 2 × 10^5^ and 10^5^ so to achieve similar running times (respectively approximately 180, 200 and 150 seconds per replicate).

DTA exhibited generally poor performance (Fig 3, and Fig. B in S1 Text), with overly narrow credible intervals. The 95% credibility intervals were not well calibrated, including the true parameter between 56%-81% of the time, compared to 80%-96% for MTT, 84%-97% for BASTA, and the theoretical target of 95% (Table 1). Furthermore, the point estimates (posterior median) were much less well correlated with the true parameter values for DTA (0.33–0.64) than for BASTA (0.51–0.85) and MTT (0.42–0.77), indicating poorer statistical efficiency.

**Table 1:** Performance of methods as a function of sampling strategy and mean migration rate in the two-population “fixed tree” scenario.

Sampling	Rate^c^	Method	Calibration^d^	Correlation^e^	RMSE^f^
Even^a^	Fast	DTA	0.56	0.58	1.83
		MTT	0.87	0.77	1.32
		BASTA	0.95	0.83	1.51
Even	Slow	DTA	0.81	0.64	1.65
		MTT	0.96	0.75	1.52
		BASTA	0.97	0.81	1.30
Uneven^b^	Fast	DTA	0.68	0.33	1.79
		MTT	0.80	0.46	2.50
		BASTA	0.84	0.70	2.08
Uneven	Slow	DTA	0.80	0.39	1.73
		MTT	0.85	0.42	2.49
		BASTA	0.88	0.51	2.29

For each combination of sampling strategy, migration rate and method, we assessed the methods’ performance across 100 replicates by recording the “true” (i.e. simulated) ratio of the migration rates *f*_1,2_/*f*_2,1_, the point estimate (posterior median) and the 95% credible interval.

^*a*^ 100 samples per population.

^*b*^ 10 samples for one population and 190 for the other.

^*c*^ total mean migration rate: fast ($\bar{f}=5.0$) or slow ($\bar{f}=0.5$).

^*d*^ proportion of replicates for which the truth fell within the 95% credible interval.

^*e*^ correlation between the truth and the point estimate.

^*f*^ root mean square error of the point estimate.

**Fig 4:**
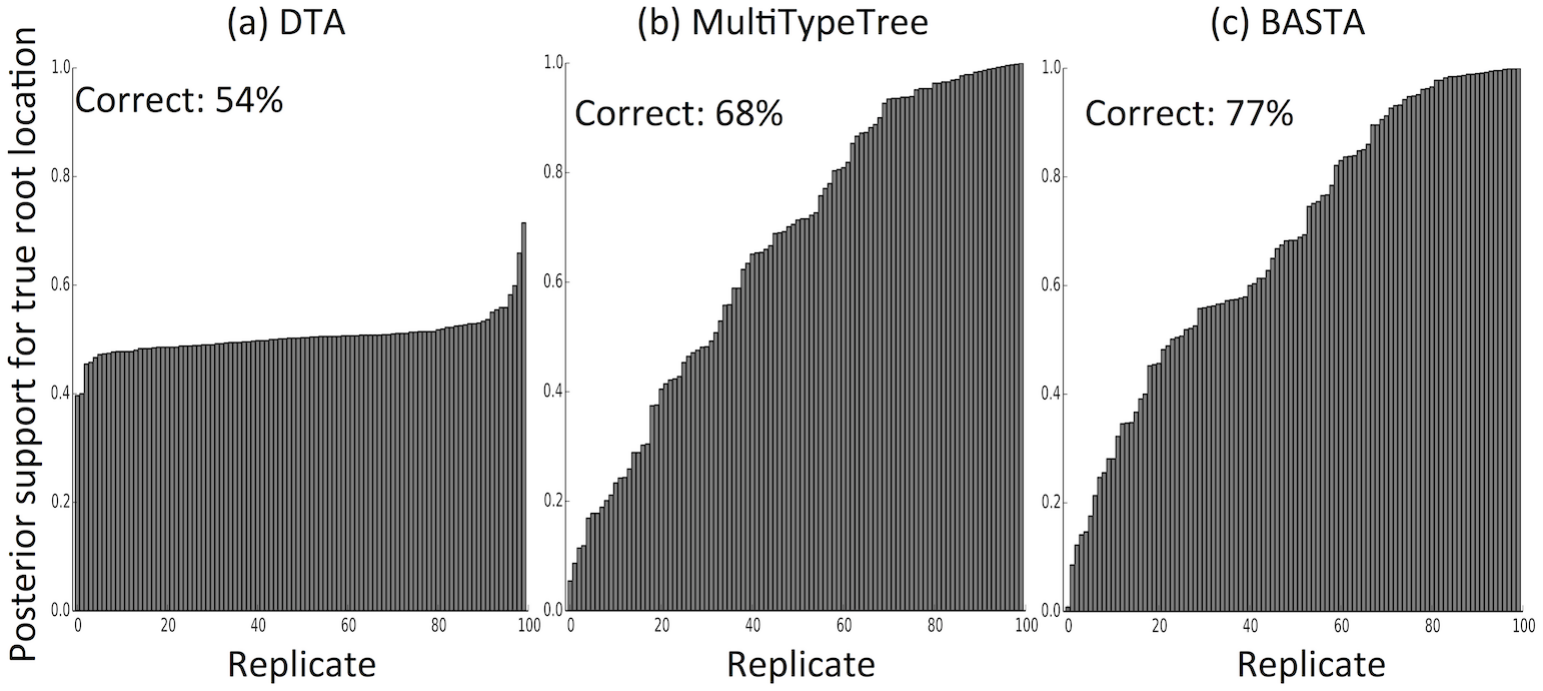
The structured coalescent improves reconstruction of ancestral subpopulations. We measured the accuracy with which ancestral subpopulations were inferred for the root (most recent common ancestor) of the genealogy using (a) DTA, (b) MTT and (c) BASTA. Each bar represents the posterior probability of the true root subpopulation (which was recorded during simulation) for an individual replicate, so taller bars represent better inference. Each bar plot is labelled with the percentage of replicates for which the point estimate was correct. Simulations were performed with two subpopulations, fixed trees, high migration rates (mean $\bar{f}=5.0$), and even sampling (100 samples per subpopulation). For each sampling strategy we simulated 100 replicates, which we ordered horizontally by posterior probability of the true root subpopulation. Number of MCMC steps for DTA, MTT and BASTA were respectively 10^6^, 2 × 10^5^ and 10^5^ so to achieve similar running times (respectively approximately 180, 200 and 150 seconds per replicate).

Poor performance was not restricted to estimating relative migration rates. The accuracy with which the location of the root (the most recent common ancestor) was estimated was 54% for DTA, compared to 68% for MTT and 77% for BASTA (Fig 4 and Fig. C in S1 Text).

Earlier methods for estimating parameters of the structured coalescent exhibited disproportionately increased computational demands with elevated migration rates due to the need to explore a larger parameter space of possible migration histories [25]. Here we found that MTT performed similarly well under different total migration rates, supporting the view that its new proposal functions represent a very considerable improvement over previous approaches [27].

We went on to assess the relative performance of the methods in a more realistic setting, when there is both phylogenetic signal and phylogenetic uncertainty (the “variable tree” scenario). This scenario is more complex as phylogenetic uncertainty makes inference more computationally demanding. All three methods account for phylogenetic uncertainty by exploring possible trees using MCMC. Again we simulated under the Continental model, this time with a 2000 bp alignment, a mutation rate of 0.01 per base per *N*_*e*_ generations, 50 samples per subpopulation and a mean migration rate of $\bar{f}=2.0$. All methods reported greater uncertainty in this setting, as expected, with DTA continuing to show weaker correlation between point estimates and the truth and severely underestimating posterior uncertainty compared to BASTA. While MTT most faithfully captured posterior uncertainty, it showed the worst correlation between point estimates and the truth, possibly reflecting a need to run it for longer than the other methods in the presence of phylogenetic uncertainty (Fig. D in S1 Text and Table 2).

**Table 2:** Performance of methods in the two-population “variable tree” scenario.

Method	Calibration^a^	Correlation^b^	RMSE^c^
DTA	0.70	0.51	1.68
MTT	0.92	0.49	2.56
BASTA	0.86	0.56	2.61

For an even sampling strategy (50 individuals per subpopulation) and moderate mean migration rate ($\bar{f}=2.0$) we assessed the methods’ performance across 100 replicates by recording the “true” (i.e. simulated) ratio of the migration rates *f*_1,2_/*f*_2,1_, the point estimate (posterior mean) and the 95% credible interval.

^*a*^ proportion of replicates for which the truth fell within the 95% credible interval.

^*b*^ correlation between the truth and the point estimate.

^*c*^ root mean square error of the point estimate.

**Fig 5:**
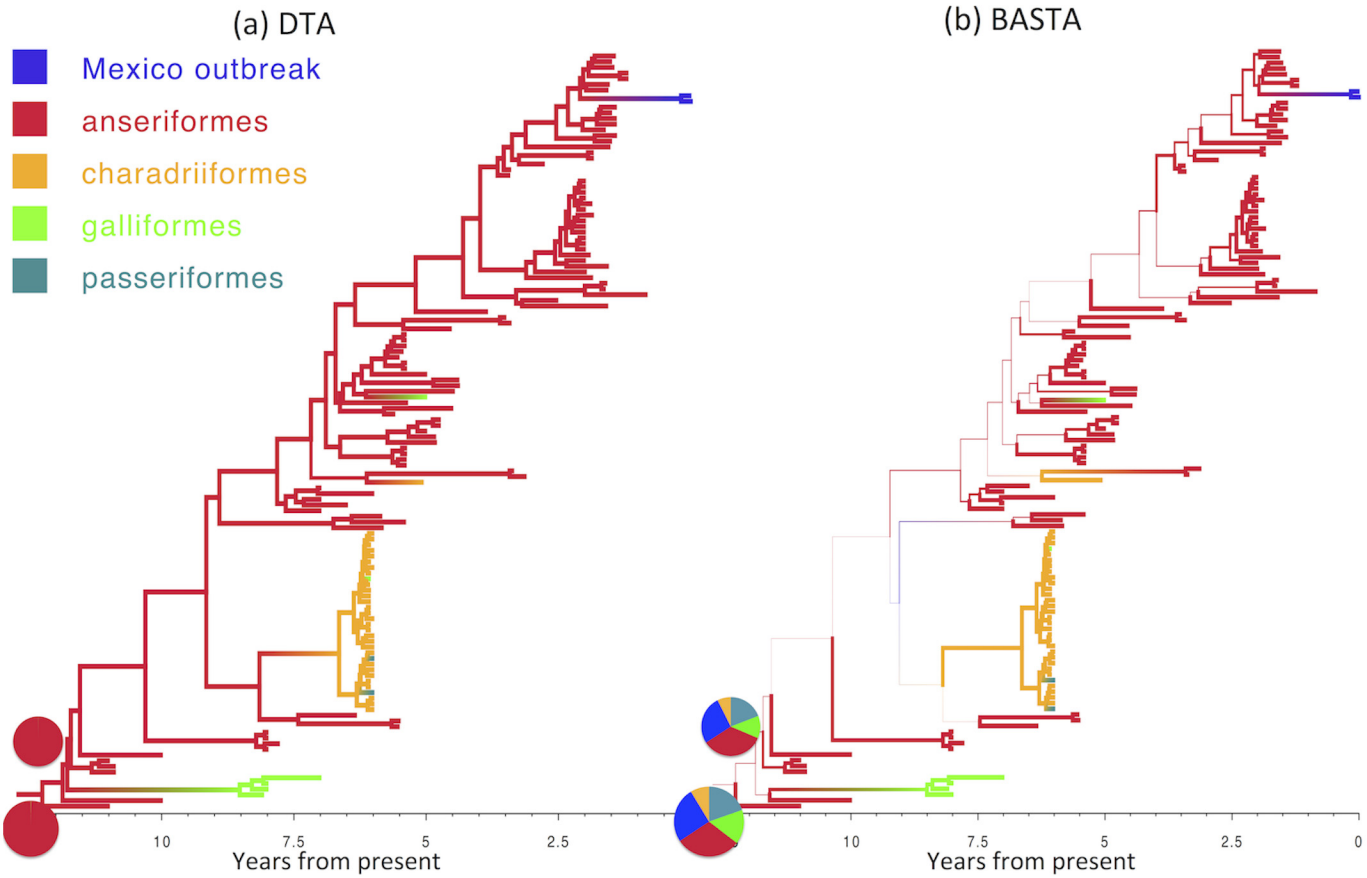
Inference of ancestral host species on the AIV dataset. Maximum clade credibility trees inferred from the AIV dataset using (a) DTA and (b) BASTA. Branch colors, as from legend, mark the inferred location at the node at the bottom of the branch, while branch width represents the posterior confidence of the inference. Although DTA and BASTA give similar inferred ancestral hosts, their interpretations are different: DTA places total confidence for most ancestral nodes, while BASTA shows very large uncertainty. Pie charts show the posterior distribution of locations inferred at two internal nodes. The scale of the axis is in number of years from present.

**Fig 6:**
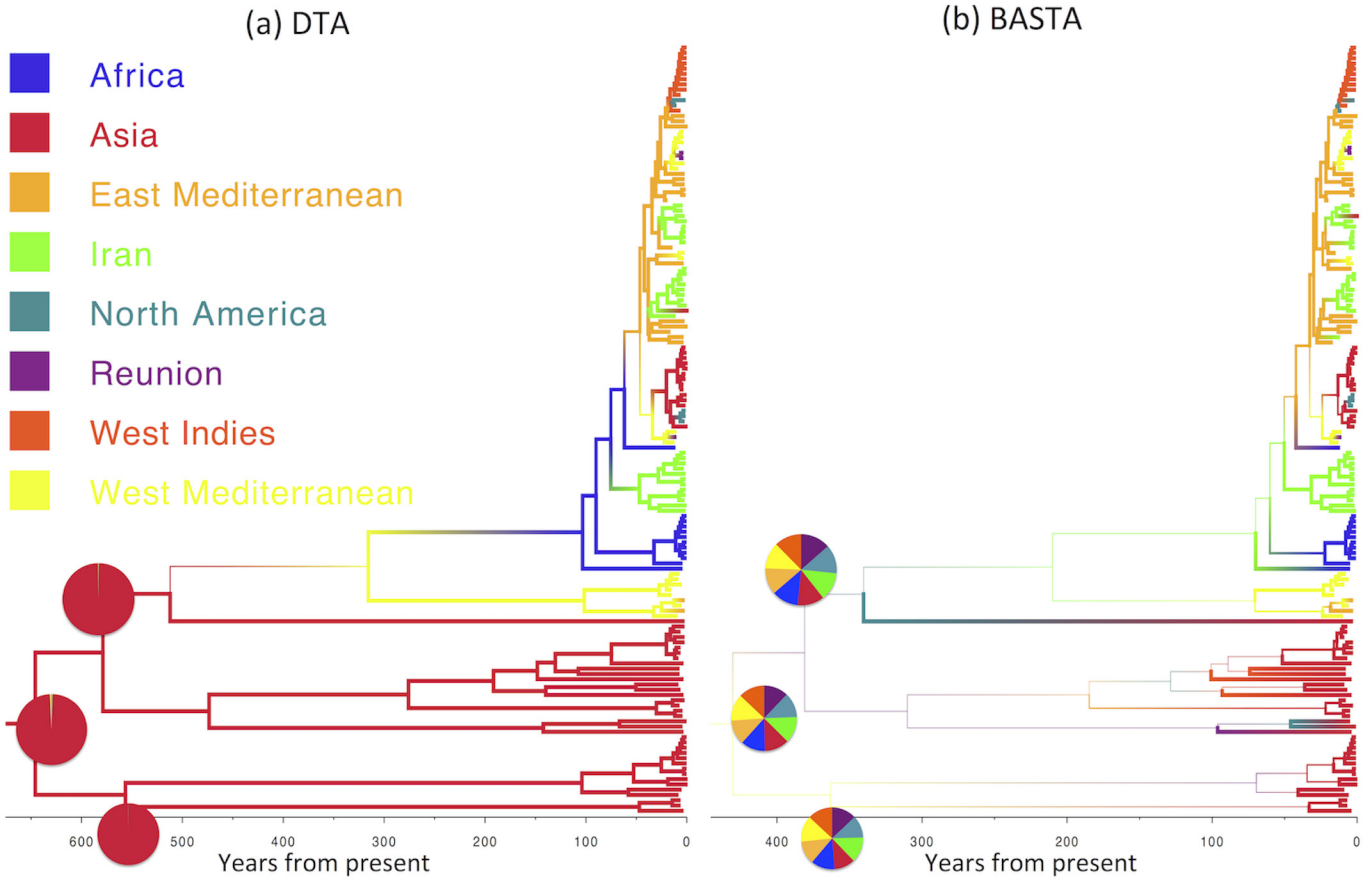
Inference of ancestral locations on the TYLCV dataset. Maximum clade credibility trees inferred from the AIV dataset using (a) DTA and (b) BASTA. Branch colors, as from legend, mark the inferred location at the node at the bottom of the branch, while branch width represents the posterior confidence of the inference. Here DTA and BASTA give again opposite interpretations: while DTA infer ancestral locations with extreme confidence, for BASTA at the same nodes all locations are equally likely. Pie charts show the posterior distribution of locations inferred at three internal nodes. The scale of the axis is in number of years from present.

The over-confidence of phylogeographic inference made by DTA appears to affect analyses of real datasets as well as simulations. We compared the results of DTA and BASTA applied to a collection of Avian Influenza Virus (AIV) sequences sampled from different avian hosts [45] and a collection of Tomato Yellow Leaf Curl Virus (TYLCV) sequences [46] sampled from different locations worldwide. For both the AIV dataset (Fig 5) and the TYLCV dataset (Fig 6), DTA reported very high confidence throughout the tree in the reconstructed ancestral subpopulations, representing host species and geographic location respectively. DTA reported posterior probabilities above 90% for ancestral reconstruction of most subpopulations (135 out of 145 internal nodes in Fig 6 and all 132 in Fig 5), even deep within the tree. In contrast, BASTA placed high confidence on ancestral subpopulation reconstruction only for internal nodes close to samples, and only the minority had subpopulation posterior probability above 90% (63 out of 145 internal nodes in Fig 6 and 61 out of 132 in Fig 5). Although we do not know the true host species and geographic locations of ancestors in these real datasets, the results of the simulations suggest that the high posterior probabilities reported by DTA could be poorly calibrated and overly confident, and that the results of BASTA are more reliable.

### BASTA is Faster than Structured Coalescent Methods

So far, we have mostly considered scenarios with just two subpopulations, for which structured coalescent methods are expected to work in a reasonable time. However, with more populations, they may be too computationally demanding for practical inference. To compare the performance of BASTA to MTT in such a scenario, we simulated an Archipelago model with eight subpopulations arranged in two clusters of four islands, with 40 samples from each island. Migration between islands in the same archipelago was assumed fast (mean 5.0) while migration between archipelagoes was 10-fold lower. To assist inference under MTT, we fixed the tree.

Both methods reported considerable uncertainty in their estimates of the migration rates and root location (Fig. G in S1 Text). However, for BASTA the MCMC algorithm reached convergence more quickly and more satisfactorily (measured by the effective sample size, ESS > 200 [49]) and in reasonable time (2 × 10^6^ MCMC steps over 1.3 × 10^4^ seconds per chain). With similar computational effort for MTT, the MCMC algorithm was far from convergence (see e.g. Figs. E and F in S1 Text for some randomly sampled replicates) with unsatisfactory estimates of the posterior distribution for most parameters (ESS < 20). These results show that not only does BASTA produce a modest but consistent improvement in calibration and statistical efficiency over MTT (see also Table 3) but it has also broader applicability to scenarios with more populations.

**Table 3:** BASTA improves migration rate estimation in the Archipelago scenario.

Method	Calibration^a^	Correlation^b^	RMSE^c^
Rate within archipelagos			
MTT	0.815	0.62	1.49
BASTA	0.95	0.69	1.33
Rate between archipelagos			
MTT	0.98	0.61	1.57
BASTA	1.00	0.67	1.47

To compare migration rate estimation between MTT and BASTA in a setting with moderate complexity we simulated an Archipelago model with two groups of four subpopulations and with 40 samples per population. 50 replicates were simulated. Performance relates to estimation of the migration rate between islands in the same archipelago and between islands in different archipelagos.

^*a*^ proportion of rates among all replicates for which the truth fell within the 95% credible interval.

^*b*^ correlation between the truth and the point estimate.

^*c*^ root mean square error of the point estimate.

These results are important for the analysis of real datasets with more than just a few subpopulations, where BASTA currently offers the only practical alternative to DTA. The number of subpopulations in the AIV and TYLCV examples are moderately high, with 5–10 host species in the former [45] (depending on pooling) and eight global locations in the latter [46]. We found that this many subpopulations challenged or exceeded the range of applicability of MTT. In the analysis of the AIV dataset, MTT required a large number of MCMC iterations to achieve convergence(Fig. I in S1 Text), while the analysis of the TYLCV data proved infeasible (Fig. J in S1 Text). In contrast, we were able to run BASTA on both datasets in less than a day (Figs 5, 6 and Fig K in S1 Text).

### Model Choice Strongly Influences Reconstruction of Ebola Transmission Dynamics

While we write, the most deadly known outbreak of Ebola virus is ongoing in West Africa. In recent work, Gire et al. [39] have collected and whole genome sequenced 99 Ebola virus samples from 78 patients. Using these and previous data, the authors have shown that all available sequences within each outbreak since 1976 cluster together phylogenetically; furthermore, divergence of lineages leading to different outbreaks usually considerably pre-dates the older outbreak. This fact and the shape of the inferred phylogeny suggest that independent zoonotic transmissions are the source of different Ebola outbreaks in humans. Ebola infections in different animals have been directly observed more than 50 times, with bats thought to be the main reservoir [50].

We addressed this subject in order to explore the potential impact of modelling considerations on epidemiological conclusions based on genetic data. We defined a highly simplified phylogeographic model with two subpopulations: the first representing human hosts, the second representing an animal reservoir. In this model, coalescence events within the human population originate from human-to-human transmission; similarly coalescence events in the animal reservoir originate from transmission between animal hosts. Migration from the animal reservoir to the human population corresponds to a zoonotic transmission. Migration from human to animal was assumed sufficiently rare to be ignored (see [50]).

Using this phylogeographic model, we investigated the effect of model choice—DTA versus structured coalescent—on the epidemiological conclusions concerning the role of zoonotic transmission in seeding human outbreaks of Ebola. We found that the two models gave diametrically opposed results.

**Fig 7:**
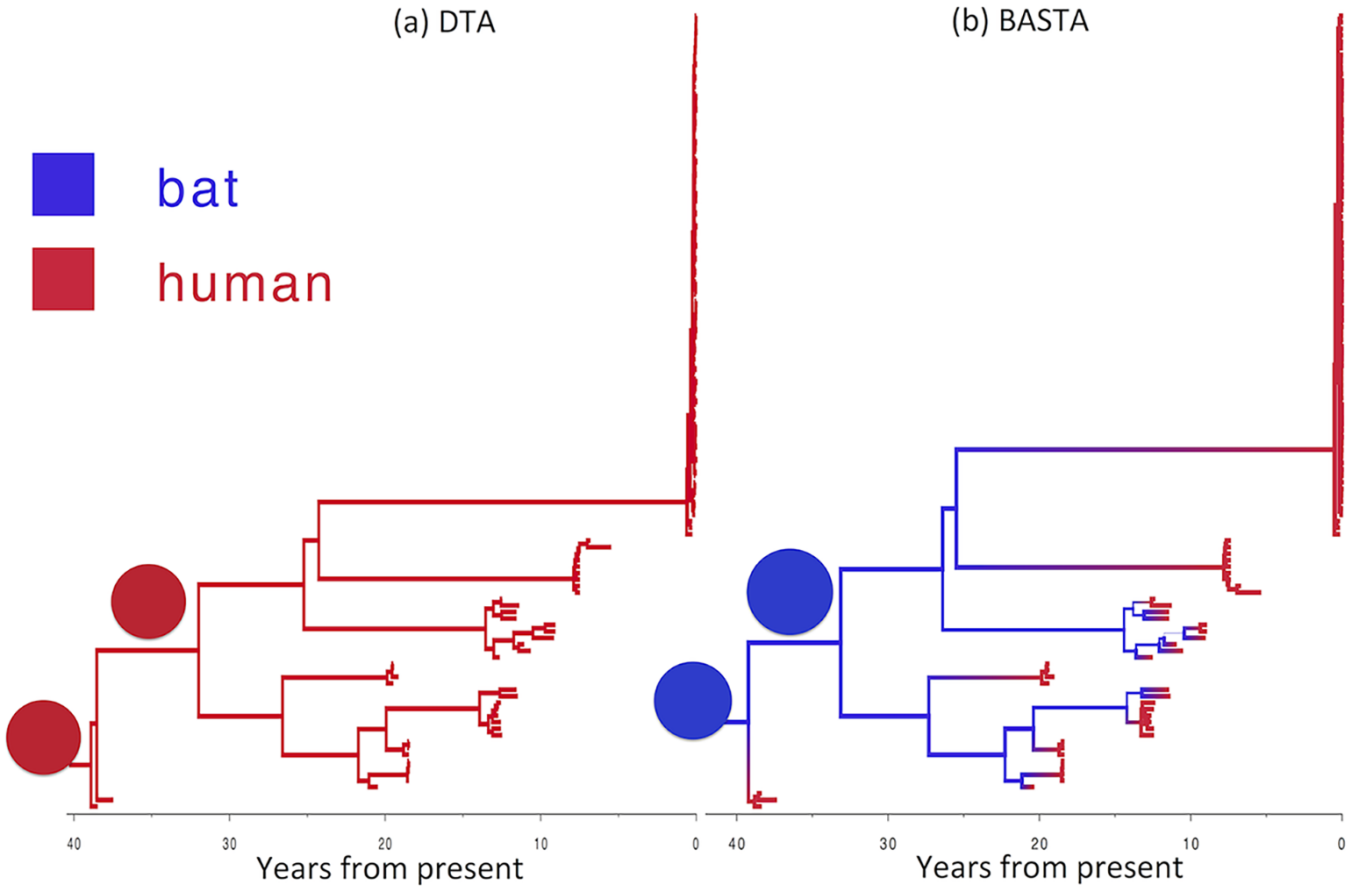
Reconstructed history of zoonosis in Ebola virus is strongly affected by the method. We reconstructed the transmission history of Ebola virus from an animal reservoir to humans using (a) DTA and (b) BASTA. Branches of the genealogy are coloured to indicate the reconstructed host species of ancestral lineages: humans (red) or bat reservoir (blue). Transitions from blue to red indicate zoonosis from an animal reservoir to humans. In the BASTA analysis, each human outbreak is precipitated by a zoonosis, whereas in the DTA analysis, no zoonosis is inferred, wrongly suggesting that the virus has persisted through undetected human-to-human transmission over the last 40 years. Branch width represents the posterior confidence on the inferred location at the node at the bottom of the branch. Pie charts (all with a single element in this stance) show the posterior distribution of locations inferred at two internal nodes.

Consistent with general understanding of the emergence of Ebola outbreaks in humans, BASTA inferred that outbreaks were seeded by independent zoonosis events from the Ebola reservoir population (Fig 7b). In keeping with this, the effective population size in the animal reservoir was inferred to be larger than in humans (median of 29.4 times larger, with 95% CI [15.7,58.1]). The most recent common ancestor of all sampled human outbreaks was inferred to have originated in the animal reservoir population with 100% posterior probability. These results were also supported by MTT.

In direct contrast, the DTA painted a very different picture of Ebola outbreak emergence that does not accord with scientific understanding. With high confidence, no zoonotic transmissions from animals to humans were inferred in the history of the sampled outbreaks (100% posterior probability, with the most recent common ancestor inferred to have occurred in the human population (Fig 7a)). Despite the implausibility of undetected human outbreaks having sustained Ebola virus in humans over four decades, DTA supported this scenario with high confidence.

To test the robustness of this result, we performed a second analysis in which we incorporated limited available Ebola sequences from bats comprising seven 265 bp partial polymerase sequences [48]. By including animal samples it was necessary to permit a very low but non-zero rate of human-to-animal migration otherwise the ancestral location of lineages ancestral to the bats would be predetermined as occurring in the animal reservoir. With the addition of samples from bats, the results were largely consistent (Fig. H in S1 Text): BASTA still inferred human outbreaks to be preceded by zoonotic transmission events from animals, with the root of the tree occurring in the animal reservoir with high probability (95%). DTA continued to erroneously infer that the majority of ancestral lineages occurred in the human population, but its confidence in this result was substantially reduced (59%).

These results illustrate the strong influence of model choice on phylogeographic inference. They demonstrate the possibility of obtaining implausible results with DTA, which may be accompanied by high posterior probabilities. Although in the case of Ebola the strength of evidence concerning the epidemiology of the disease is more than sufficient to disregard the discrete trait analysis out of hand, it demonstrates the potential to produce highly misleading inference when independent epidemiological understanding is scarce.

## Discussion

Phylogeography has rapidly gained prominence in a wide range of settings where it can quantify historical patterns of migration from just genetic data and sampling locations. In the context of infectious disease epidemics, phylogeographic methods have been used to infer transmission rates and patterns of spread even in the complete absence of reliable epidemiological information (see e.g. [9–11, 51]). Yet, these methods have only been partially tested and compared. Here, through a combination of simulations based on explicit process-driven population genetics models and real data analysis, we showed that different methods exhibit dramatic differences in their inference properties, and these differences have a direct influence on biological interpretation.

While discrete trait analysis (DTA) is extremely fast and accounts for phylogenetic uncertainty, it has difficulty accurately estimating migration rates even with as few as two subpopulations. In particular, DTA is sensitive to the relative sampling intensity of subpopulations, such that the sampling strategy adopted can influence the results, particularly when migration rates are high and genetic data are sparse. We reiterate that we have assessed the performance of DTA as a model of migration, and not in the context of the evolution of discrete traits (such as genetic or phenotypic traits), for which DTA was originally developed. MTT, on the other hand, was robust to sampling strategy, produced less biased and less noisy parameter estimates, and produced well-calibrated reports of parameter uncertainty.

Together with other methods based on the structured coalescent, MTT has the additional advantage over DTA of explicitly modelling, and therefore being able to estimate and account for, differences in the sizes of subpopulations. MTT proved useful even when migration rates were elevated, where previous structured coalescent-based methods showed convergence problems. However, we found that when moderately many subpopulations were analysed (we simulated eight, but [27] suggest not to exceed four), MTT can suffer convergence issues. To deal with this problem, we proposed a new approach, BASTA, based on an approximation to the structured coalescent similar to those of [37] and [38]. BASTA approximately integrates over all possible migration histories rather than explicitly parameterizing them and exploring them with MCMC, thereby considerably reducing the computational requirements of the method. Not only did this approach show appreciable improvements in accuracy with respect to MTT with just two populations, but it was easily able to analyse eight subpopulations in 3–4 hours, whereas analysis of this many subpopulations was beyond the reach of MTT in feasible time.

In the future, we will explore possible extensions of the model to cases with many demes, for example in patient-to-patient transmission inference, or in a stepping-stone island model [52]. In these scenarios, the technique of matrix exponentiation might prove too computationally demanding, and approaches based on shorter time subintervals, as in [37, 38], could be more efficient, particularly when the migration matrix is sparse.

In applications to real AIV and TYLCV datasets, we showed that BASTA could be used in cases of up to ten sub-populations, where MTT struggles to converge. We found that DTA reported much more confidence—and on the basis of simulations, over-confidence—in the inferred reconstruction of ancestral subpopulations than BASTA, which simulations found to be well calibrated, indicating that the methods produce substantial differences of interpretation in phylogeography studies.

Finally, analysing real data from Ebola outbreaks in humans we underlined the importance of model choice, by showing that different models can lead in practice to completely different results. In fact, diametrically opposite phylogeographic patterns were estimated using DTA versus structured coalescent-based methods. We recommend that users exercise caution in choosing phylogeographic models, and we point out that methods based on the structured coalescent are in general more reliable in modelling migration, although also more computationally demanding. The fact that the three approaches considered here are all implemented in the same phylogenetic package (BEAST2) is a considerable advantage, as it is possible to run and compare different methods while installing a single piece of software and using similar formats.

## Supporting Information

S1 Text Supplementary Text: contains computational details of BASTA, Table A, and Figures A-K. (PDF)

S1 Dataset Simulated and real datasets used in this study. Files are in xml format, so to make our analyses easily replicable in BEAST.
(ZIP)
