# Supplementary material for: New Routes to Phylogeography: A Bayesian Structured Coalescent Approximation
Source: PLoS Genet. 2015 Aug 12;11(8):e1005421. doi: 10.1371/journal.pgen.1005421 (PMC4534465; doi:10.1371/journal.pgen.1005421)

New Routes to Phylogeography: a Bayesian Structured Coalescent Approximation

S1 Text

Computational Details of BASTA - Eq. 10

Calculating Eqs. 11 and 12 requires similar steps to Felsenstein's pruning algorithm, and also has similar computational demands. We therefore do not focus on its details here. Instead we show how we calculate the coalescent rates (Eq. 10), and in particular, the sum

$$\sum_{d\in D}\sum_{l\in\Lambda}\sum_{l'\in\Lambda,l'\neq l} P_{l,t,d}P_{l',t,d}\frac{1}{\theta_d}$$

for a given time t, a given set of extant lineages Λ, and given the probabilities $\boldsymbol{P}_{l,t}$ and $\boldsymbol{P}_{l',t}$. For brevity, from now on we ignore the time index t. If the expected number of lineages in a deme d is represented as $\mathbb{E}(n_d) := \sum_{l\in\Lambda} P_{l,d}$, we have:

$$\sum_{d\in D}\sum_{l\in\Lambda}\sum_{l'\in\Lambda,l'\neq l} P_{l,d}P_{l',d}\frac{1}{\theta_d} = \sum_{d\in D}\left[\left(\sum_{l\in\Lambda}\sum_{l'\in\Lambda} P_{l,d}P_{l',d}\frac{1}{\theta_d}\right) - \left(\sum_{l\in\Lambda} P_{l,d}P_{l,d}\frac{1}{\theta_d}\right)\right] = \sum_{d\in D}\frac{1}{\theta_d}\left[\mathbb{E}(n_d)\mathbb{E}(n_d) - \sum_{l\in\Lambda} P_{l,d}P_{l,d}\right].$$

Let us call $S_d = \sum_{l\in\Lambda} P_{l,d}P_{l,d}$. Calculating S_d requires $O(|\Lambda|)$ time and is needed for each deme and twice for each coalescent event. So, if n denotes the number of samples, the total cost of computing S_d for the whole tree is approximately $O(n^2\cdot|D|)$. Updating S_d after a coalescent or sampling event is trivial and negligible in time. Calculating $\mathbb{E}(n_d)$ is also faster, as we can use the same procedure in Eq. 11 which avoids the sum over lineages, giving a required time of $\approx O(n\cdot|D|^2)$. Updating $\mathbb{E}(n_d)$ after coalescence and sampling events is trivial and fast. Calculating the exponential of the migration rate matrix used in Eq. 11 is required once per event, for a total computational cost $< O(n\cdot|D|^3)$. Lastly, while Eq. 12 requires negligible time, Eq. 11 has computational cost $\approx O(|D|^2)$, that repeated over all lineages and over all events, brings to a total cost of $\approx O(n^2\cdot|D|^2)$, which is the computational bottleneck of BASTA (generally $n \gg |D|$).

To further reduce the computational time, we adopt a caching technique that consists in using the same vectors for lineages that have undergone the same history since sampling (including same sampling location). If many leaves are sampled at the same time, this leads to important savings, but in the worst scenario the total computational demand remains $\approx O(n^2\cdot|D|^2)$.

Tables

Table A. Summary of model assumptions

	Discrete Trait Analysis	Structured Coalescent
**Sampling scheme**	Deme sampling intensity proportional to deme prevalence at sampling time.	Any.
**Deme prevalence**	Variable through time, deme prevalence can drift and demes can temporarily disappear from global population.	All demes are constantly present through time at constant prevalence, each deme with its own size.
**Tree shape**	Migration does not affect tree shape, instead standard coalescence is assumed.	Effective population sizes and migration rates affect coalescence rates and tree shape.

Figures

Figure A. DTA is also inherently biased at low migration rates. To test for inherent sampling bias, we again analysed a dataset containing just sampling locations, but no genetic information using (a) DTA, (b) MTT and (c) BASTA. For a method robust to sampling, the posteriors (green and blue distributions) should be unchanged from the prior (pink distribution). However, DTA treats the sampling process as informative about migration parameters, and this leads to an overestimation of low migration rates. The blue and green posterior distributions correspond respectively to even sampling (100 samples per subpopulation) and uneven sampling (10 and 190 samples per location). The mean migration rate was $\bar{f} = 0.1$. Each plot is obtained from ten merged posteriors of independent MCMC runs each of 5×10^6 iterations.

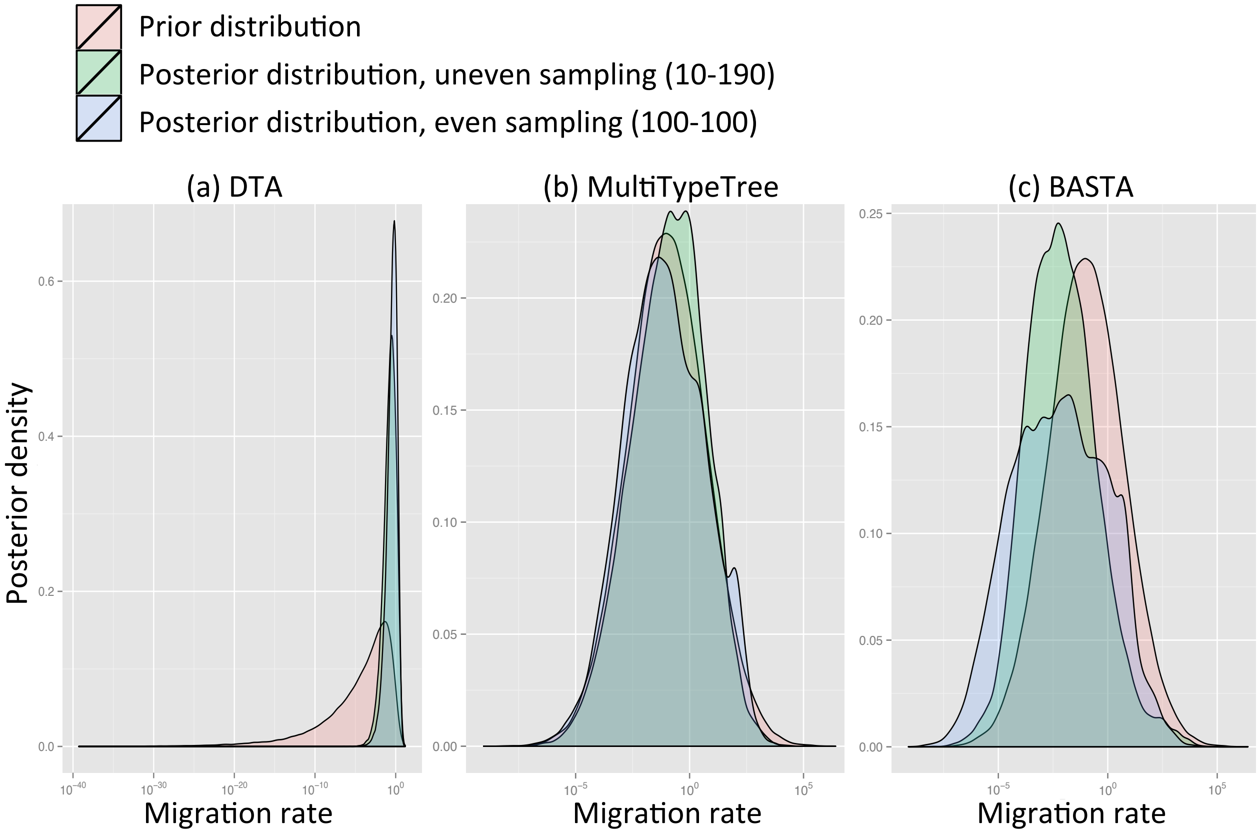

Figure B. Comparison of rate estimation with two populations and fixed tree. To test the accuracy of the 95% credible intervals produced by (a,d,g) DTA, (b,e,h) MTT and (c,f,i) BASTA, we simulated and analysed 100 datasets under the two-population "Continental" model. We provided the true genealogy to BEAST2, as if it were estimated without error; in this scenario methods are expected to give the best accuracy. The migration rates between the subpopulations were simulated for each dataset from a prior distribution, and we compared the "true" ratio $f_{1,2}/f_{2,1}$ (horizontal axis) to the point estimate (posterior median; vertical axis, points) and 95% credible interval (2.5 and 97.5 percentiles; error bars). The results confirm a weaker correlation between the truth and the point estimates for DTA, compared to MTT and BASTA, indicating worse statistical efficiency. The percentage of datasets in which the 95% credible intervals contained the truth revealed that DTA was poorly calibrated compared to MTT, BASTA and the theoretical target of 95%. The dashed line indicates the hypothetical optimal estimate. Number of MCMC steps for DTA, MTT and BASTA are respectively 10^6, 2×10^5 and 10^5 so to achieve similar running times (respectively approximately 180, 200 and 150 seconds per replicate). (a-c) 100 samples from each population, and low mean migration rate $\bar{f} = 0.5$. (d-f) 10 samples from one population and 190 from the other, and high mean migration rate $\bar{f} = 5.0$. (g-i) 10 samples from one population and 190 from the other, and low mean migration rate $\bar{f} = 0.5$.

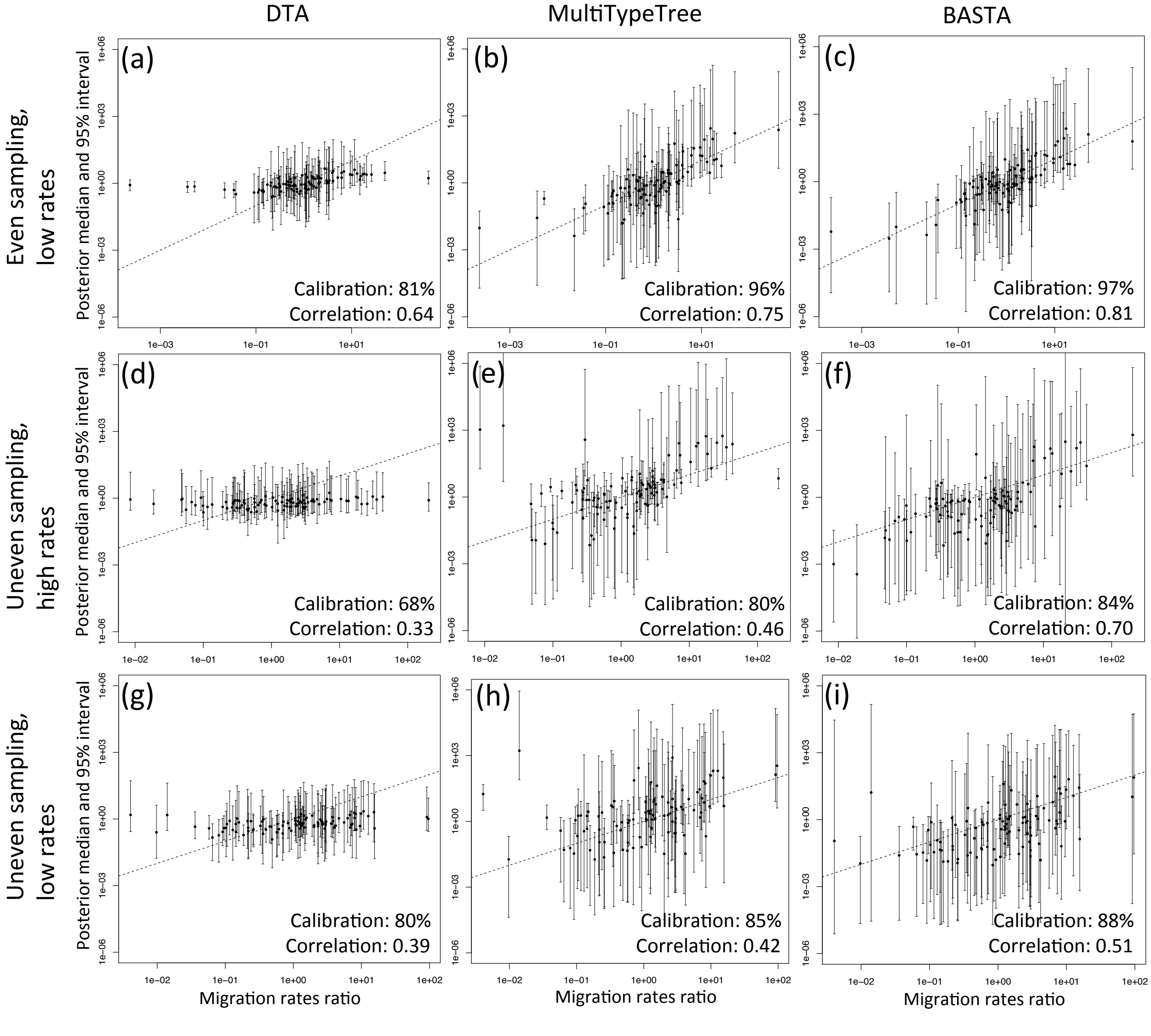

Figure C. Ancestral subpopulation reconstruction. We measured the accuracy with which ancestral subpopulations were inferred for the root (most recent common ancestor) of the genealogy using (a,d,g) DTA, (b,e,h) MTT, (c,f,i) BASTA. Each bar represents the posterior probability of the true root subpopulation (which was recorded during simulation) for an individual replicate, so taller bars represent better inference. Each bar plot is labelled with the percentage of replicates for which the point estimate was correct. Simulations were performed with two subpopulations and fixed trees. For each setting we simulated 100 replicates, which we ordered horizontally by posterior probability of the true root subpopulation. Number of MCMC steps for DTA, MTT and BASTA were respectively 10^6, 2×10^5 and 10^5 so to achieve similar running times (respectively approximately 180, 200 and 150 seconds per replicate). (a-c) 100 samples from each population, and low mean migration rate $\bar{f} = 0.5$. (d-f) 10 samples from one population and 190 from the other, and high mean migration rate $\bar{f} = 5.0$. (g-i) 10 samples from one population and 190 from the other, and low mean migration rate $\bar{f} = 0.5$.

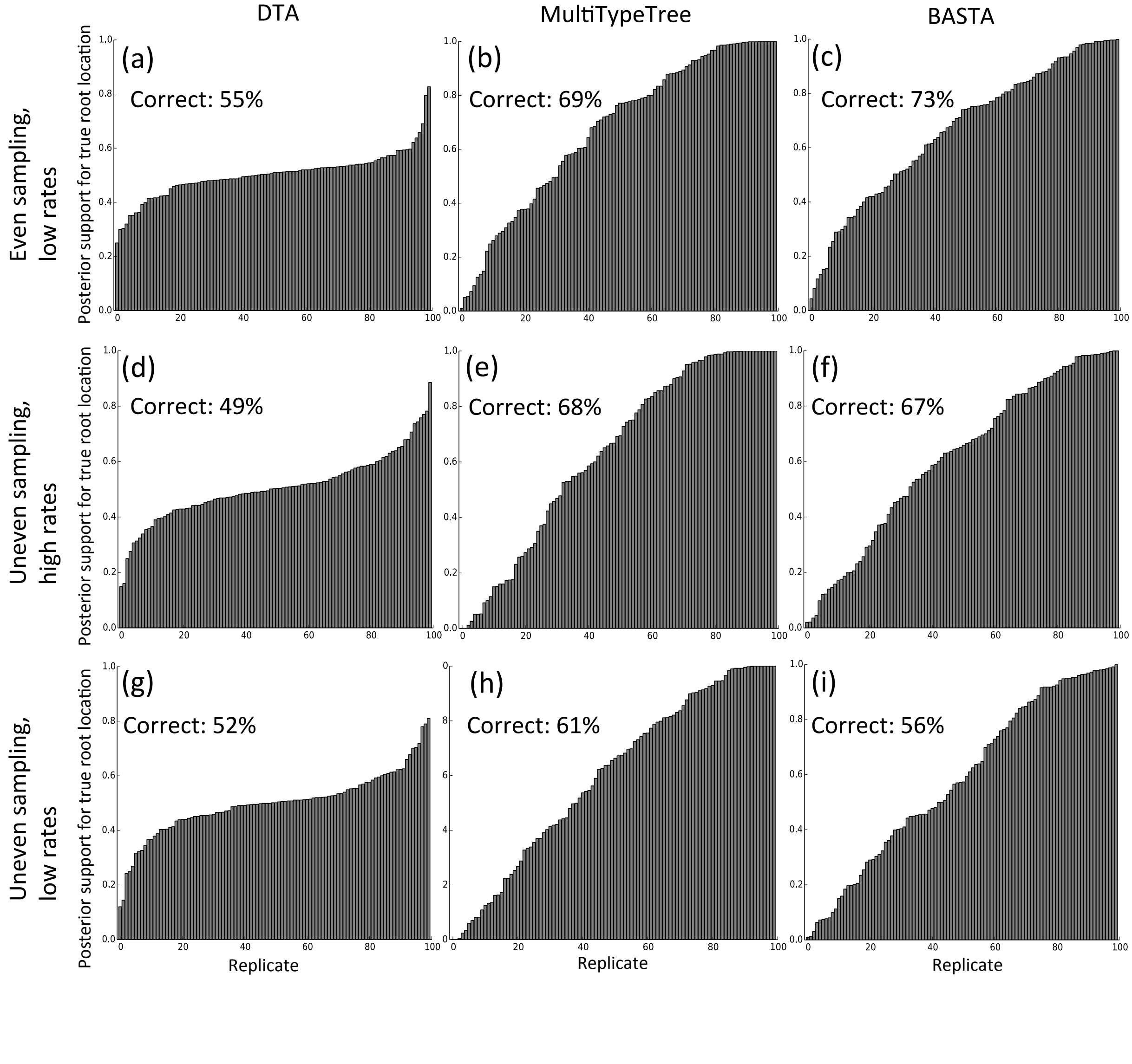

Figure D. Phylogenetic uncertainty hinders phylogeographic reconstruction. In many cases only short sequences are available for phylogenetic reconstruction, and this provides limited phylogenetic signal. We simulated this scenario by generating 2000 bp alignments from which phylogenies are inferred together with phylogeographic parameters. The migration rates between the subpopulations were simulated for each dataset from a prior distribution, and in (a-c) we compared the "true" ratio $f_{1,2}/f_{2,1}$ (horizontal axis) to the point estimate (posterior median; vertical axis, points) and 95% credible interval (2.5 and 97.5 percentiles; error bars), while in (d-f) each bar represents the posterior probability of the true root subpopulation for an individual replicate. For estimation we used (a,d) DTA, (b,e) MTT, and (c,f) BASTA. In this scenario, MTT and BASTA still provide mostly better inference than DTA, although all methods are negatively affected by the phylogenetic uncertainty. We performed 50 total replicates under intermediate mean migration rate $\bar{f} = 2.0$ and with 50 samples from each population. Number of MCMC steps for DTA, MTT and BASTA were respectively 2×10^7, 2×10^7 and 10^7 for running time of respectively approximately 2000, 7000 and 4500 seconds per replicate.

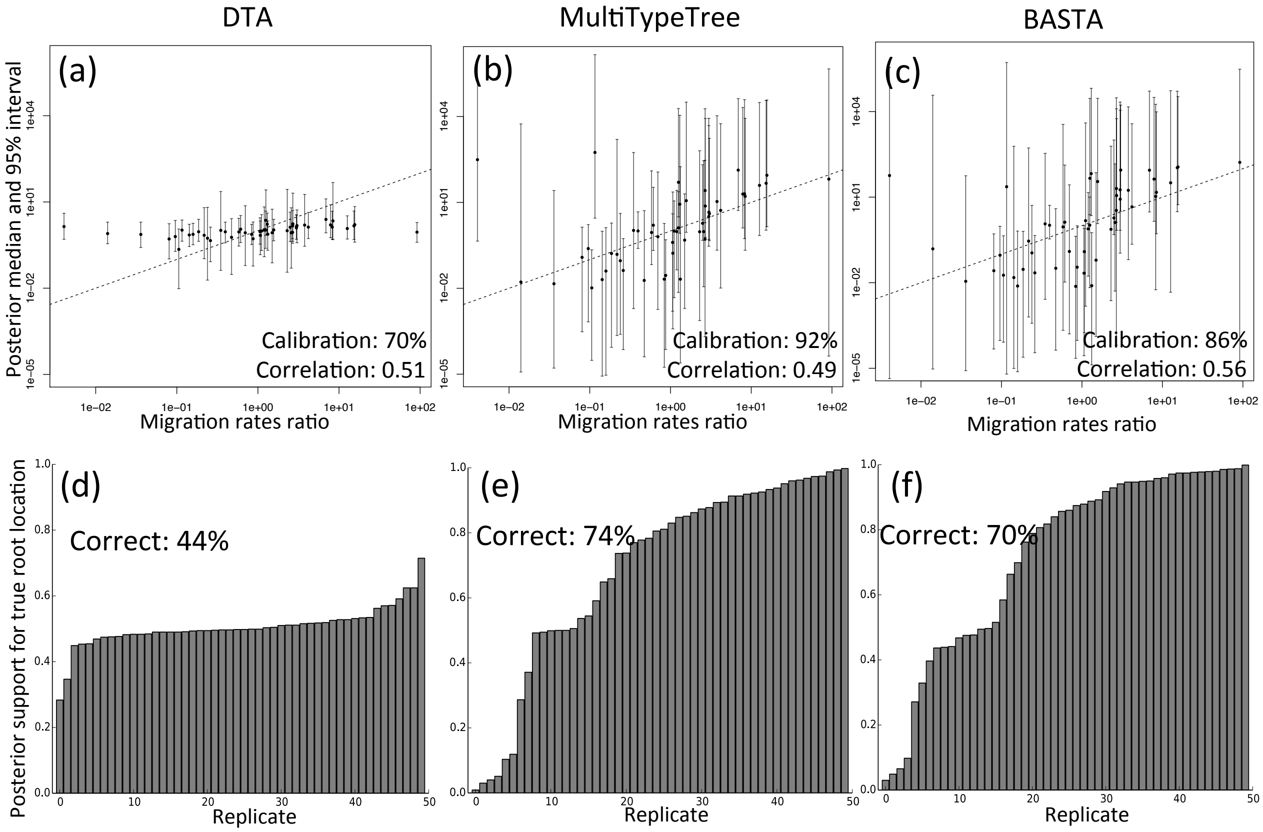

Figure E. BASTA has broader applicability than MTT. In the analysis of datasets simulated under a moderately complex eight-population Archipelago model, BASTA always efficiently explored the posterior distribution of the parameters in acceptable time, while MTT, with comparable computational resources, never achieved convergence. With these plots we show the traces of the posterior probability density (a,b) and migration rates (c,d) on the Y axis, over the MCMC steps (X axis) in one random replicate. (a) Posterior probability density with MTT. (b) Posterior probability density with BASTA. (c) Migration rate from deme 1 to 2 with MTT. (d) Migration rate from deme 1 to 2 with BASTA. Similar plots for further replicates are found in Fig.F. For these simulations we used fixed trees and 40 samples for each of the eight populations.

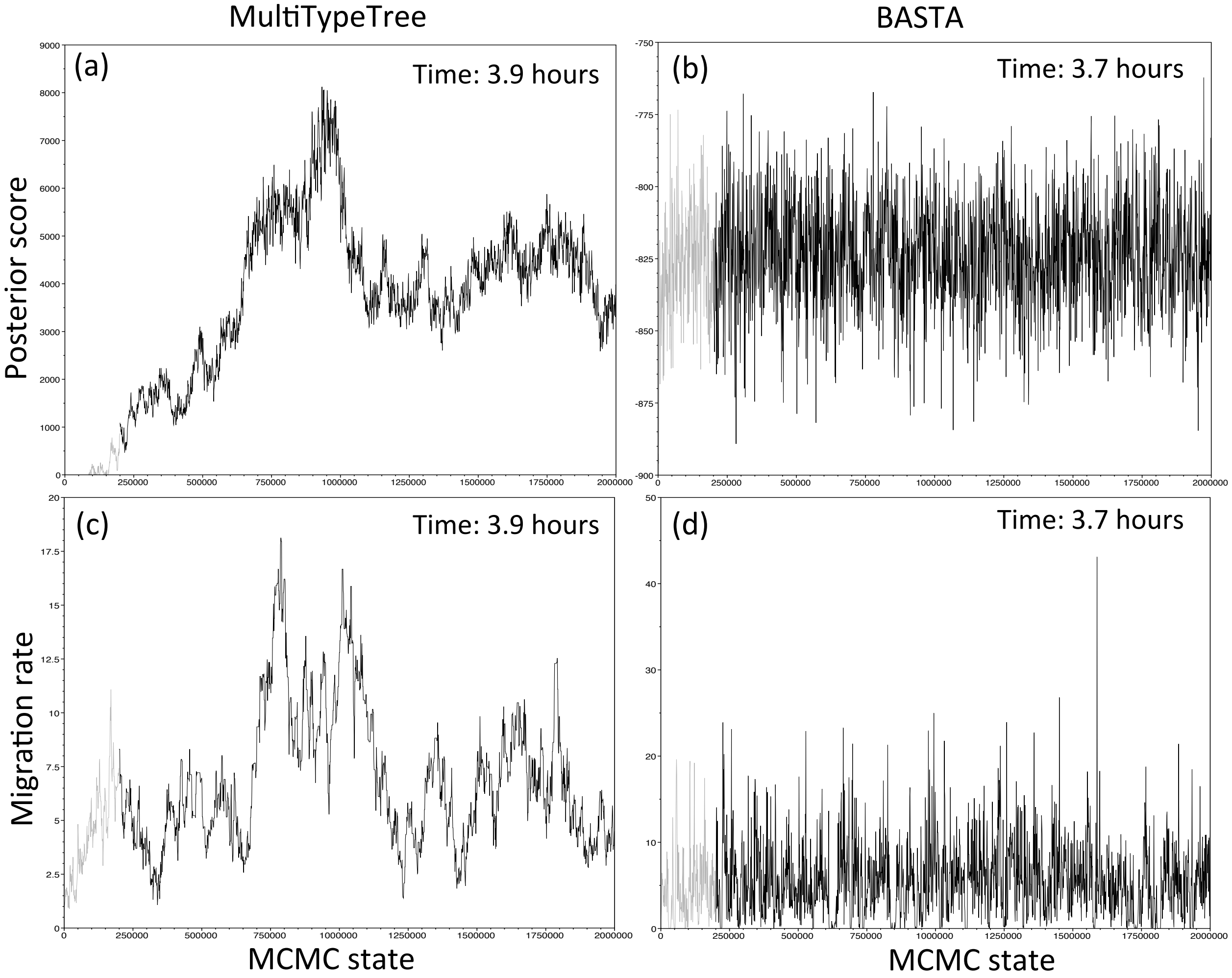

Figure F. Other examples of BASTA and MTT posterior traces with eight populations. Here we show further examples that under a moderately complex eight-population model, BASTA always efficiently explored the posterior distribution of the parameters in acceptable time, while MTT did not achieve convergence. With these plots we show the traces of the posterior probability density (a,b) and migration rates (c,d) on the Y axis, over the MCMC steps (X axis) in one random replicate. (a,c,e,g) Posterior probability density with MTT. (b,d,f,h) Posterior probability density with BASTA. We used fixed trees and 40 samples for each of the eight populations.

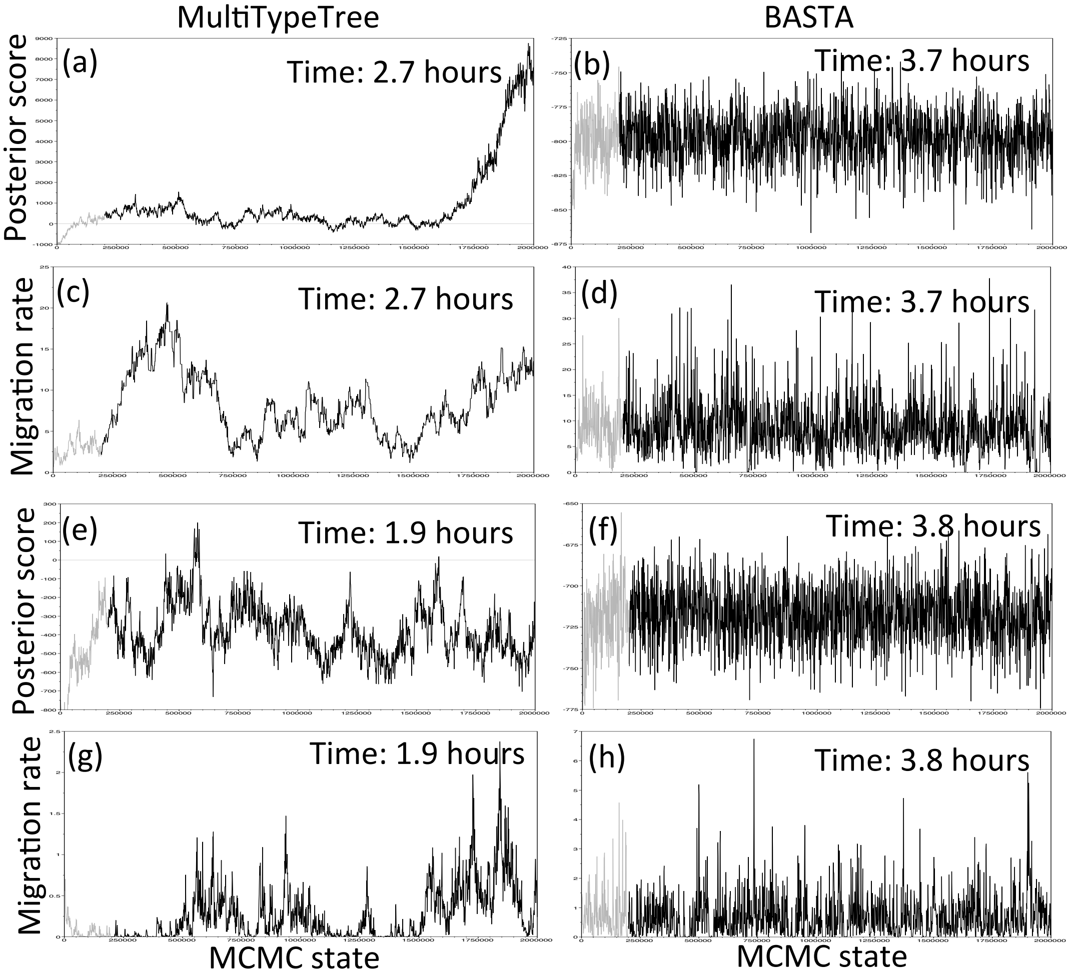

Figure G. High uncertainty in estimation with eight populations. In the analysis of datasets simulated under a moderately complex eight-population Archipelago model, both BASTA and MTT show very large uncertainty in phylogeographic estimation. We used fixed trees, 40 samples for each of the eight populations, and 50 replicates in total. The migration rates between the subpopulations were simulated for each dataset from a prior distribution, and in (a,b) we compared the "true" ratio $f_{1,2}/f_{2,1}$ (horizontal axis) to the point estimate (posterior median; vertical axis, points) and 95% credible interval (2.5 and 97.5 percentiles; error bars), while in (c,d) each bar represents the posterior probability of the true root subpopulation for an individual replicate. For estimation we used (a,c) MTT and (b,d) BASTA. Number of MCMC steps for MTT and BASTA were 2×10^6 for running time of respectively approximately 1.5×10^4 and 1.3×10^4 seconds per replicate.

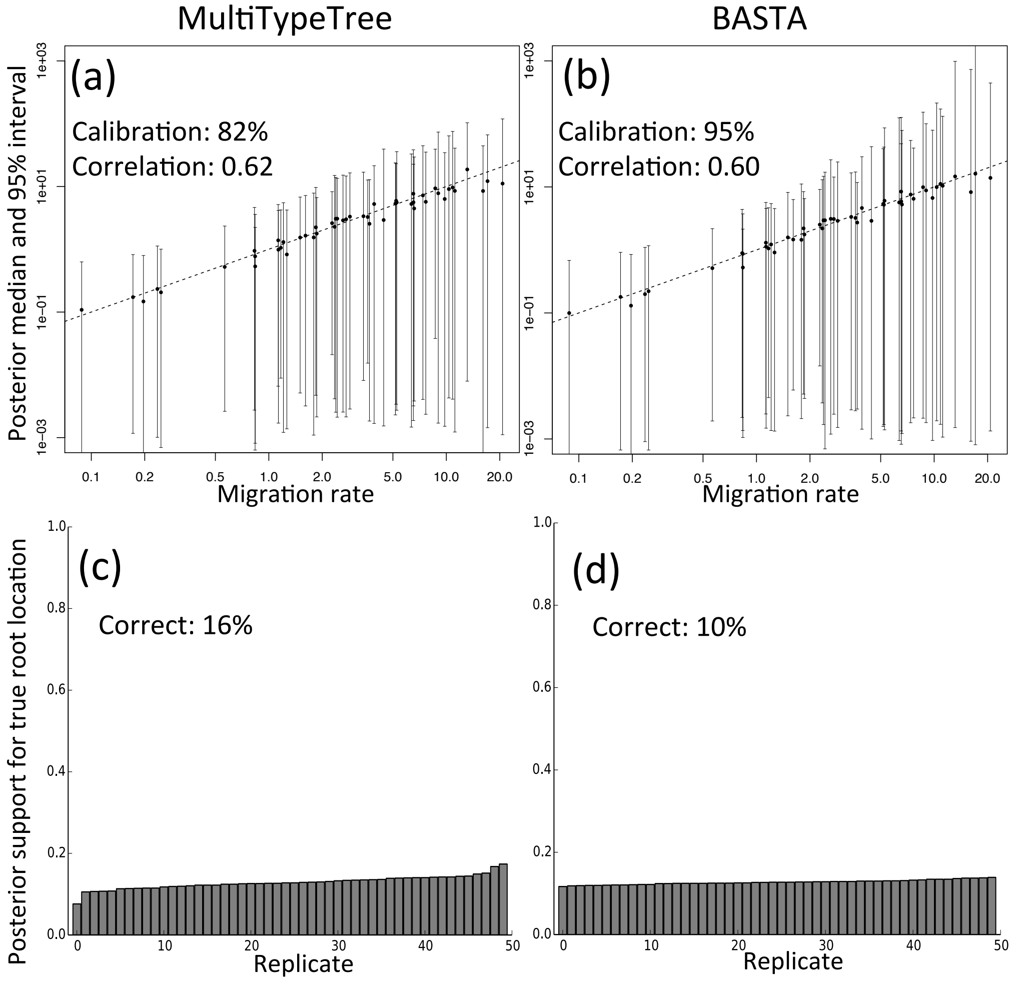

Figure H. Different zoonotic transmission histories for the extended Ebola Dataset. Maximum clade credibility trees inferred using (a) DTA and (b) BASTA. In red we show nodes of the phylogeny inferred to be in human host, while in blue are nodes inferred to be in bat reservoirs. The scale of the axis is in number of years from present. Here we included the 265 bp region from six bat samples. Differently from the main text, here we allow migration from human to bat reservoir at very low rate (10^{-5} times lower than from bat to human), otherwise the root and most internal nodes would be necessarily inferred in bats by any model. We see that here, even with the addition of Ebola samples from bats, DTA still infers human ancestral location, differently from BASTA. Branch width represents the posterior confidence of the inferred location at the node at the bottom of the branch. Pie charts show the posterior distribution of locations inferred at two internal nodes.

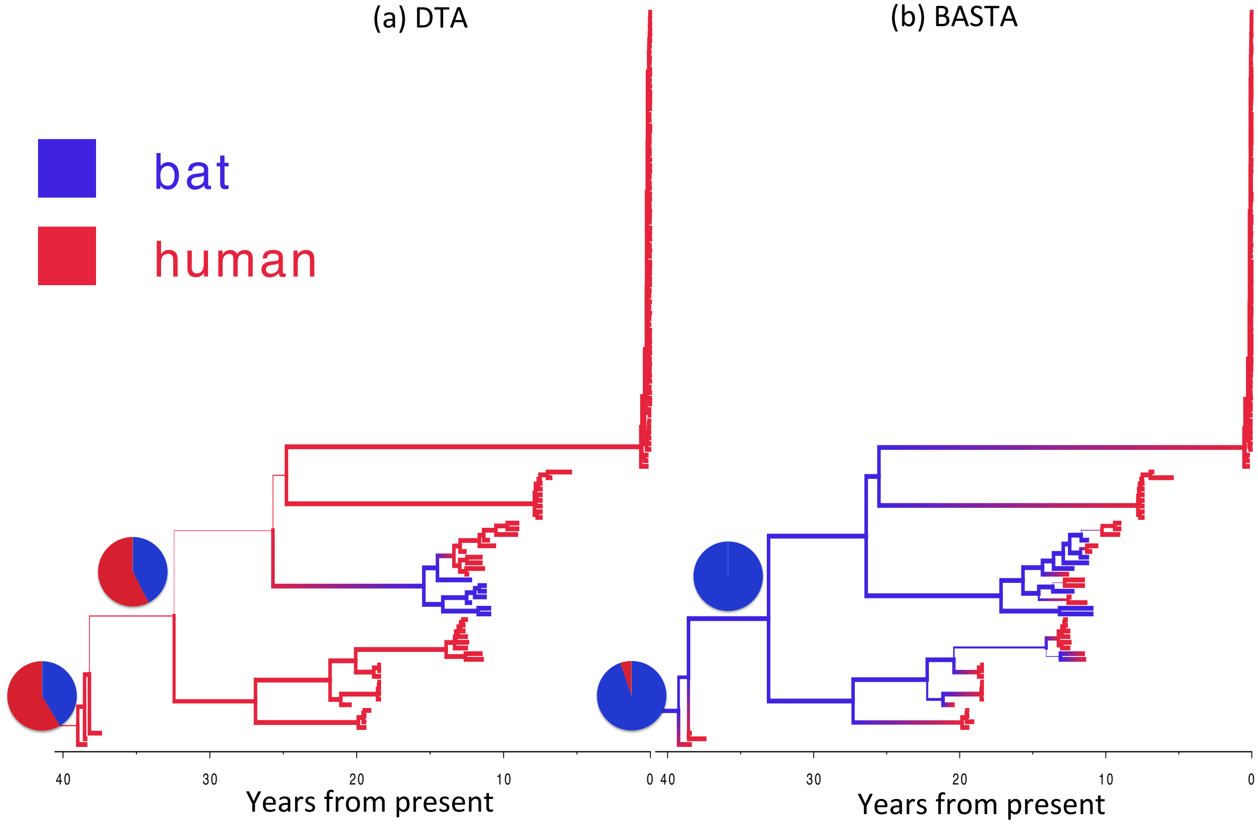

Figure I. BASTA achieves faster convergence than MTT on the AIV dataset. On the AIV dataset with either five or ten defined populations, BASTA (first and third row) efficiently explored the posterior distribution of the parameters in acceptable time, while MTT (second and fourth row), with comparable computational resources, did not achieved convergence. Plots show the traces of the posterior probability density (left column), one migration rate (central column), and effective population size (right column) on the Y axis, over the MCMC steps (X axis). All traces consist of 2×10^7 MCMC iterations (X axes show the step number). In the top half are results for the AIV dataset with five populations, while in the bottom half are results for the AIV dataset with ten populations.

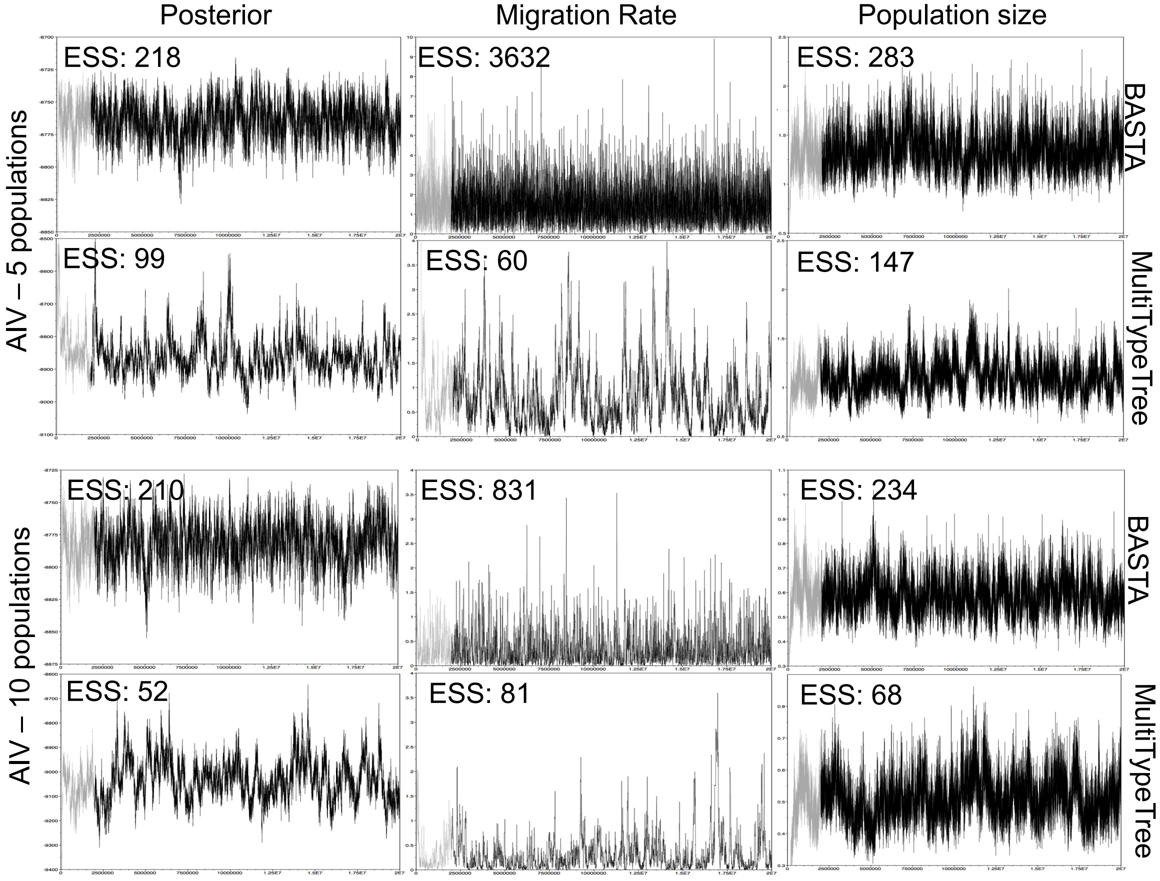

Figure J. BASTA achieves faster convergence than MTT on the TYLCV dataset. On the TYLCV dataset BASTA efficiently explored the posterior distribution of the parameters in acceptable time (< 1 day), while MTT, with larger computational resources, did not approach convergence. Plots show the traces of the posterior probability density (left column), one migration rate (central column), and effective population size (right column) on the Y axis, over the MCMC steps (X axis). BASTA (top row) was run for 5×10^7 MCMC iterations (X axes show the step number), which took less than one day, while MTT (bottom row) was halted after slightly more than 1 day.

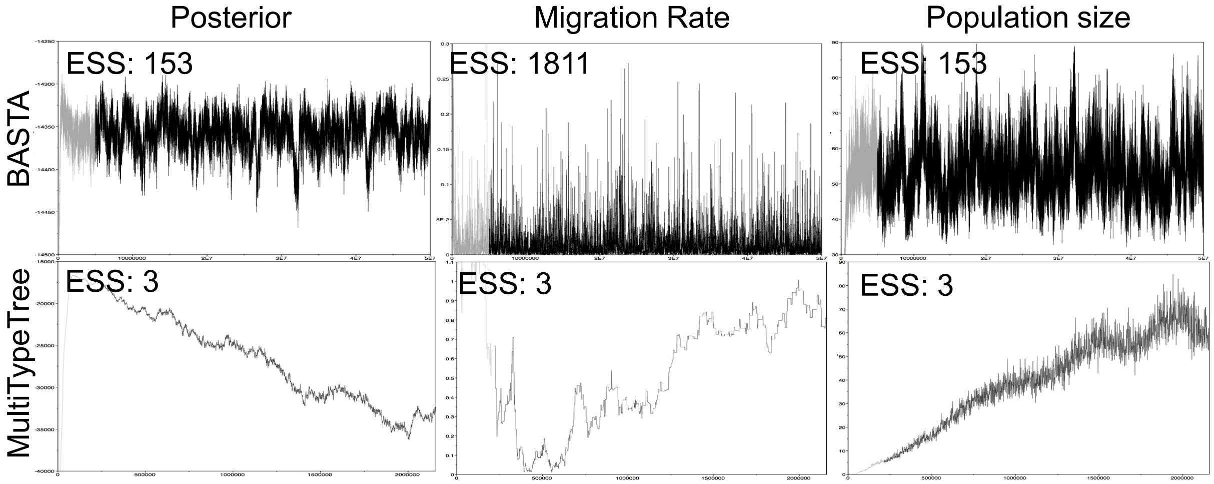

Figure K. Inference of ancestral host species on the AIV dataset with specific host species. Maximum clade credibility trees inferred from the AIV dataset using (a) DTA and (b) BASTA. Branch colors, as from legend, mark the inferred host at the node at the bottom of the branch, while branch width represents the posterior confidence of the inference. DTA and BASTA give different results, with DTA inferring ancestral locations with some confidence, while for BASTA at the same nodes all locations are equally likely. The pie charts show the posterior distribution of locations inferred at the root. The scale of the axis is in number of years from present.

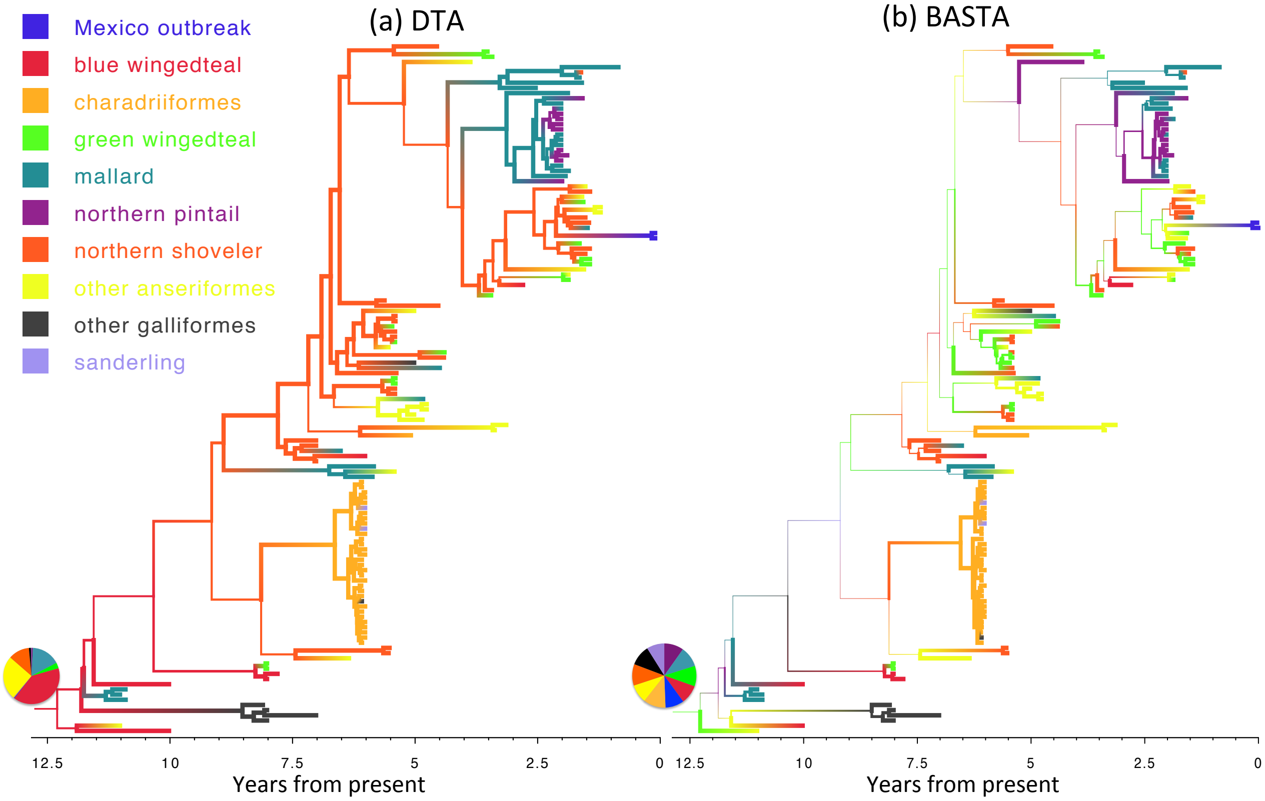

Supplement: S1 Text — (PDF) [file pgen.1005421.s001.pdf]
